# 2D arrays of hollow carbon nanoboxes: outward contraction-induced hollowing mechanism in Fe–N–C catalysts†

**DOI:** 10.1039/d4sc01257g

**Published:** 2024-05-17

**Authors:** Xiaokai Song, Xiaoke Wang, Jiamin Wei, Shenghua Zhou, Haifeng Wang, Jiali Lou, Yaqi Zhang, Yuhai Liu, Luyao Zou, Yingji Zhao, Xiaoqian Wei, Sameh M. Osman, Xiaopeng Li, Yusuke Yamauchi

**Affiliations:** a Institute of Advanced Functional Materials for Energy, School of Chemistry and Chemical Engineering, Jiangsu University of Technology Changzhou 213001 China weijiamin@jsut.edu.cn; b State Key Laboratory for Modification of Chemical Fibers and Polymer Materials, College of Materials Science and Engineering, Donghua University Shanghai 201620 China xiaopeng.li@dhu.edu.cn; c Department of Materials Process Engineering, Graduate School of Engineering, Nagoya University Nagoya 464-8603 Japan; d Chemistry Department, College of Science, King Saud University P. O. Box 2455 Riyadh 11451 Saudi Arabia; e Australian Institute for Bioengineering and Nanotechnology (AIBN), The University of Queensland Brisbane Queensland 4072 Australia y.yamauchi@uq.edu.au; f Department of Plant & Environmental New Resources, College of Life Sciences, Kyung Hee University 1732 Deogyeong-daero, Giheung-gu Yongin-si Gyeonggi-do 17104 South Korea

## Abstract

Maximizing the utilization efficiency of monatomic Fe sites in Fe–N–C catalysts poses a significant challenge for their commercial applications. Herein, a structural and electronic dual-modulation is achieved on a Fe–N–C catalyst to substantially enhance its catalytic performance. We develop a facile multi-component ice-templating co-assembly (MIC) strategy to construct two-dimensional (2D) arrays of monatomic Fe-anchored hollow carbon nanoboxes (Fe-HCBA) *via* a novel dual-outward interfacial contraction hollowing mechanism. The pore engineering not only enlarges the physical surface area and pore volume but also doubles the electrochemically active specific surface area. Additionally, the unique 2D carbon array structure reduces interfacial resistance and promotes electron/mass transfer. Consequently, the Fe-HCBA catalysts exhibit superior oxygen reduction performance with a six-fold enhancement in both mass activity (1.84 A cm^−2^) and turnover frequency (0.048 e^−^ site^−1^ s^−1^), compared to microporous Fe–N–C catalysts. Moreover, the incorporation of phosphorus further enhances the total electrocatalytic performance by three times by regulating the electron structure of Fe–N_4_ sites. Benefitting from these outstanding characteristics, the optimal 2D P/Fe-HCBA catalyst exhibits great applicability in rechargeable liquid- and solid-state zinc–air batteries with peak power densities of 186 and 44.5 mW cm^−2^, respectively.

## Abstract 2

A structural and electronic dual-modulation is achieved on 2D arrays of hollow carbon nanoboxes, exhibiting exceptional performance in rechargeable zinc–air batteries.

## Graphical abstract

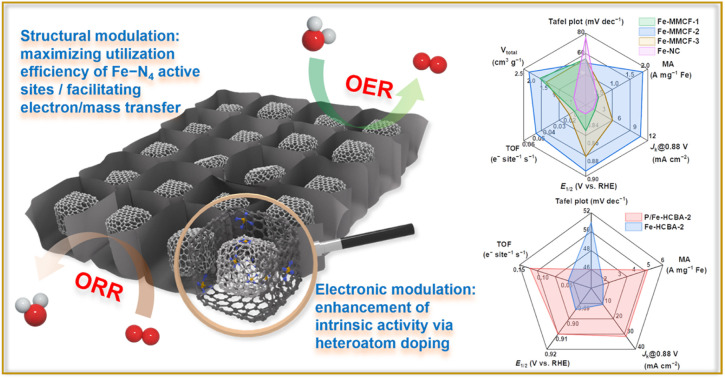

## Introduction

Electrochemical oxygen reduction reaction (ORR) and oxygen evolution reaction (OER) assume a pivotal role in the field of sustainable energy conversion and storage devices, such as zinc–air batteries (ZABs).^1,2^ To date, platinum group metal (PGM) catalysts are used as the most efficient catalysts for ORR and OER.^3,4^ However, suffering from their scarcity and unsustainability, it is of great significance to develop non-PGM ORR catalysts with high activity and durability. Among them, iron–nitrogen–carbon (Fe–N–C) catalysts are the most promising alternatives to PGM catalysts due to their unique electronic structure and theoretical maximum atomic utilization efficiency.^5–7^ Furthermore, heteroatom doping (*e.g.*, S, B, and P), either in bonding or non-bonding configurations to the single metal sites, can further optimize the electronic structure of Fe–N_*x*_ sites, enhancing the intrinsic activity of Fe–N–C catalysts.^8–11^ Additionally, the overall electrocatalytic performance is determined not only by the intrinsic activity of active sites but also by the number and utilization efficiency of accessible active sites. Since the ORR or OER occurs at the electrode/electrolyte interface, maximizing accessibility to Fe–N_*x*_ sites is significantly influenced by the size, geometry, and specifically, the pore nature of the catalyst.^12–15^ Zeolite imidazolate frameworks (ZIFs) have been commonly employed as ideal templates for the synthesis of catalysts incorporating Fe–N_*x*_ sites.^16–19^ Nevertheless, direct pyrolysis of ZIF-8 nanoparticles inevitably yields a porous structure rich in microporous regions that hinders contact between reactants and active sites, while weakening the utilization efficiency of buried Fe–N_*x*_ active sites within microporous carbon, thus inhibiting mass transfer efficiency and mass-specific activity of the catalyst.^20–22^ Therefore, it is imperative to design hierarchically porous structures incorporating both hollow cavities and/or highly open nanoarchitectures for Fe–N–C catalysts.

Hollow carbon is typically prepared using the hard-templating method, which involves employing templates such as silica spheres (SiO_2_),^23^ polystyrene (PS) spheres,^24^ ZnO spheres,^25,26^ or CdS spheres.^27^ This synthesis strategy allows for the preparation of either monodisperse zero-dimensional (0D) hollow carbon nanoparticles or three-dimensional (3D) macroporous carbon materials. For example, using SiO_2_ as a template and ZIF as a carbon source, 0D hollow carbon nanoparticles or 3D interconnected carbon frameworks can be successfully synthesized.^28,29^ Furthermore, 3D-ordered macroporous carbon nanocrystals can be prepared through the PS colloidal crystal templating method.^30^ However, these hard-templating methods often involve complex synthesis processes and environmentally unfriendly template removal steps. Achieving two-dimensional (2D) monolayered hollow carbon materials, with a thickness equivalent to that of a single nanoparticle, remains a significant challenge due to the difficulty in the self-assembly of template nanoparticles into 2D monolayered assemblies.^31^

Alternatively, ZIF nanocrystals can serve as sacrificial templates to prepare hollow carbon nanoarchitectures, such as 0D hollow carbon polyhedra,^32^ one-dimensional (1D) carbon nanofibers,^33^ and 3D carbon aerogels,^34^ through an outward contraction stress-induced synthesis approach. In this process, another carbon source (*e.g.*, polyvinyl pyrrolidone (PVP), polyacrylamide (PAN), polydopamine (PDA)) is coated on the surface of ZIF nanocrystals using various coating methods to selectively form thin carbon shells. These shells induce the decomposition of ZIF nanocrystals, resulting in the formation of large cavities.^35^ However, the fabrication of ZIF-templated 2D monolayered hollow carbon nanoarchitecture has been rarely reported and is mainly hindered by the difficulties in assembling ZIF nanocrystals and additional carbon sources into 2D monolayers.^36^

**Fig. 1:**
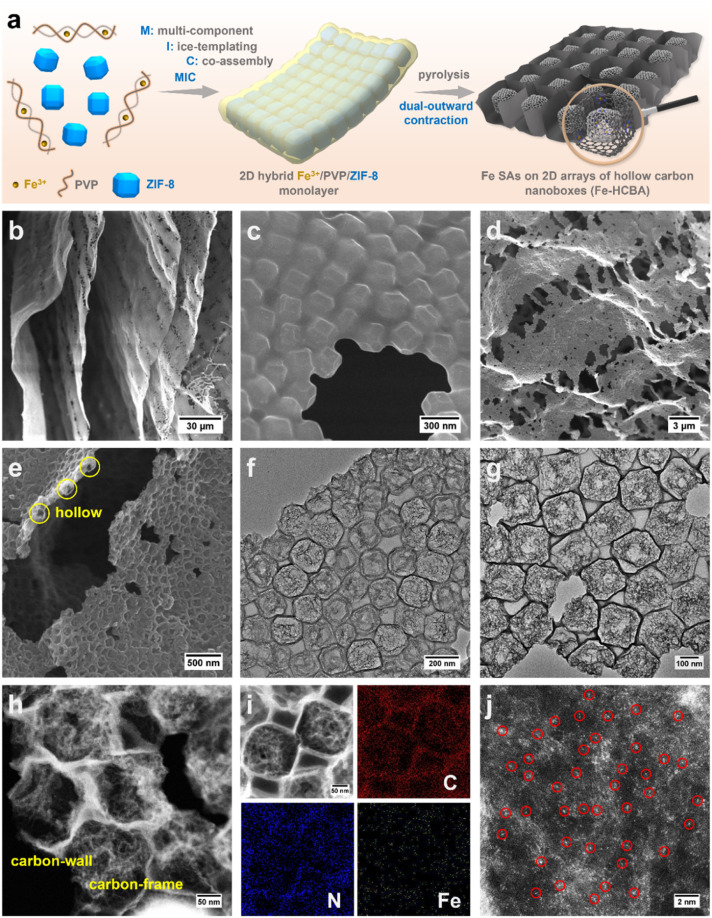
(a) Schematic illustration of the synthetic process for Fe-HCBA. (b and c) SEM images of Fe^3+^/PVP/ZIF-8-2. (d and e) SEM, (f and g) TEM, (h and i) Scanning TEM (STEM) images and the corresponding elemental mapping of Fe-HCBA-2, and (j) atomic-resolution HAADF-STEM of Fe-HCBA-2.

Leveraging a comprehensive understanding of the mechanism underlying hollowing in ZIF-derived hollow carbon and capitalizing on the advantages of ice-templating self-assembly strategy for large-scale fabrication of 2D metal–organic framework monolayer superstructures, we meticulously engineer 2D arrays of hollow carbon nanoboxes anchored with monatomic Fe (Fe-HCBA). Through structural and electronic dual-modulation, we significantly enhance its catalytic performance to meet the requirements for application in ZABs. A facile and efficient multi-component ice-templating co-assembly (MIC) approach is utilized to prepare 2D Fe^3+^/PVP/ZIF-8 hybrid monolayers, which are subsequently transferred into 2D Fe-HCBA *via* a dual-outward contraction stress-induced hollowing mechanism (Fig. 1a). During the pyrolysis process, ultrathin caron walls are preferentially formed around ZIF-8 polyhedra in the monolayer, inducing outward contraction stress (*F*_outward_1). Simultaneously, as the carbon wall develops, the initially isolated ZIF-8 units approach each other closely, resulting in a second outward contraction stress (*F*_outward_2) originating from the interconnected facets. By harnessing the synergy between *F*_outward_1 and *F*_outward_2 forces, robust 2D Fe-HCBA composed of well-arranged hollow carbon nanoboxes with ultrathin carbon walls and highly porous carbon frames in the cavities are constructed. Furthermore, the incorporation of Fe^3+^ facilitates the generation of abundant Fe–N_4_ active sites on both carbon walls and frames. The outward contraction hollowing mechanism-based pore engineering not only enlarges the physical surface area and pore volume to maximize the exposure of active sites but also increases the electrochemically accessible surface area by 2.7 times to promote utilization efficiency. The unique 2D monolayered carbon array architecture effectively promotes electron transfer, reduces interfacial resistance, and facilitates mass transfer in the three-phase reaction, thereby exhibiting excellent catalytic performance. Moreover, the doping of phosphorus regulates the electron structure of Fe–N_4_ active sites, further enhancing the intrinsic activity of the P/Fe-HCBA catalyst and leading to satisfactory performance in rechargeable ZABs with both liquid and solid-state electrolytes.

## Results and discussion

### Fabrication and structural characterization

The MIC of ZIF-8 colloids, PVP, and Fe^3+^ with varying mass ratios results in the formation of a series of hybrid Fe^3+^/PVP/ZIF-8 monolayers, designated as Fe^3+^/PVP/ZIF-8-*x* (for details, see ESI†). Low magnification scanning electron microscope (LM-SEM) images demonstrate the large-scale 2D thin nanosheet morphology of Fe^3+^/PVP/ZIF-8-2 (Fig. 1b and S1a†). Scanning electron microscope (SEM) and transmission electron microscope (TEM) images show that truncated rhombic dodecahedral ZIF-8 nanocrystals are well-arranged and coated with PVP in a monolayer of Fe^3+^/PVP/ZIF-8-2 (Fig. 1c and S1b–g†). Elemental mapping images show that the Fe element is uniformly distributed on the surface and in the gap region among ZIF-8 polyhedra (Fig. S1h†). Subsequently, pyrolysis of Fe^3+^/PVP/ZIF-8-2 was conducted in a N_2_ atmosphere at 900 °C to obtain Fe-HCBA-2, and its 2D large nanosheet-like morphology is well maintained (Fig. S2a†). SEM images exhibit the carbon monolayer of well-arranged carbon nanoboxes (Fig. 1d, e and S2b–d†). TEM images clearly show that hollow carbon nanoboxes with distinct carbon walls are tightly interconnected in the monolayered architectures (Fig. 1f, g and S2e–g†). Scanning TEM (STEM) image provides further evidence for the ultrathin carbon walls and highly porous carbon frames within the Fe-HCBA-2 (Fig. 1h). Elemental mapping confirms the homogeneous dispersion of Fe, C, and N elements in Fe-HCBA-2 (Fig. 1i). Notably, atomic-resolution HAADF-STEM image of Fe-HCBA-2 (Fig. 1j) illustrates that the bright dots on the carbon matrix can be identified as atomically dispersed Fe single atoms (SAs).

**Fig. 2:**
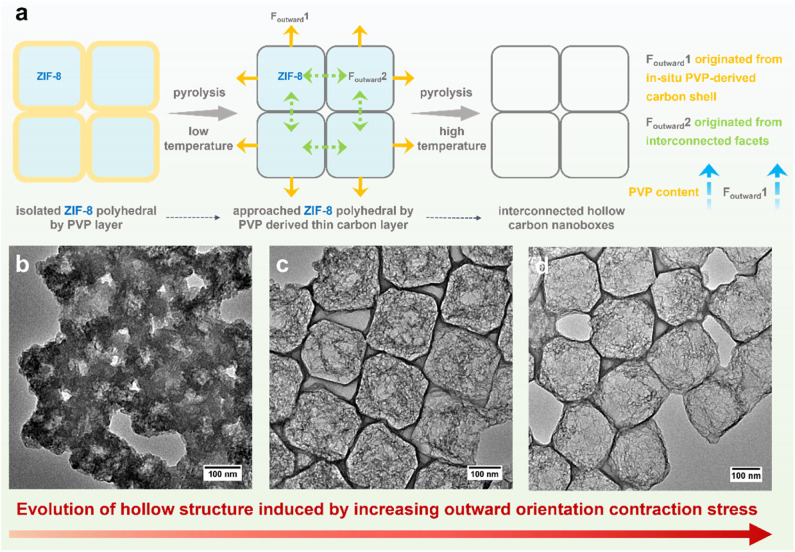
(a) Schematic illustration of dual-outward contraction mechanism for fabrication of 2D arrays of hollow carbon nanoboxes. TEM images of (b) Fe-HCBA-1, (c) Fe-HCBA-2, and (d) Fe-HCBA-3.

To understand the evolutionary process of hollow architecture, Fe-HCBA-1 and Fe-HCBA-3 were prepared from Fe^3+^/PVP/ZIF-8-1 and Fe^3+^/PVP/ZIF-8-3, respectively. The successful co-assembly of ZIF-8 with increasing amounts of PVP is evidenced by the gradual reduction in peak intensity observed in powder X-ray diffraction (PXRD) patterns and decreased Brunauer–Emmett–Teller (BET) surface areas calculated from N_2_ sorption measurements of Fe^3+^/PVP/ZIF-8-*x* (Fig. S3 and S4†). SEM and TEM images for Fe-HCBA-1 and Fe-HCBA-3 are presented in Fig. S5 and S6.† A dual-outward contraction stress-induced hollowing mechanism is illustrated in Fig. 2a. At low PVP loading amounts, the *in situ* formed carbon layer may exhibit insufficient thickness to provide a robust interfacial interaction with ZIF-8 polyhedron, resulting in a weak outward contraction stress *F*_outward_1, leading to an underdeveloped hollow structure of Fe-HCBA-1 (Fig. 2b). In this case, *F*_outward_2 predominantly governs the hollowing process of Fe-HCBA-1. With increasing PVP content, the thickened carbon walls offer enhanced interaction and significantly amplify *F*_outward_1, which synergistically contributes along with *F*_outward_2 to well-developed hollow architectures with distinct carbon walls and inner carbon frames (Fig. 2c). Upon further increase in PVP content, the overall outward contraction stress is further augmented while maintaining a constant thickness of the carbon wall; however, the inner carbon frames are almost absence due to the full decomposition of ZIF-8. This results in the formation of 2D arrays of fully hollow carbon nanoboxes (Fig. 2d). This observation strongly suggests that the evolution of the Fe-HCBA-*x* is governed by the synergy between *F*_outward_1 and *F*_outward_2. The temperature-dependent hollowing process was also investigated. With an increase in pyrolysis temperature from 500 to 900 °C, the decomposition of ZIF-8 polyhedra gradually occurs starting from the center point, resulting in the formation of highly porous carbon frames within the preferentially formed carbon nanoboxes (Fig. S7†). This observation further confirms the dual-outward contraction stress-induced hollowing mechanism.

**Fig. 3:**
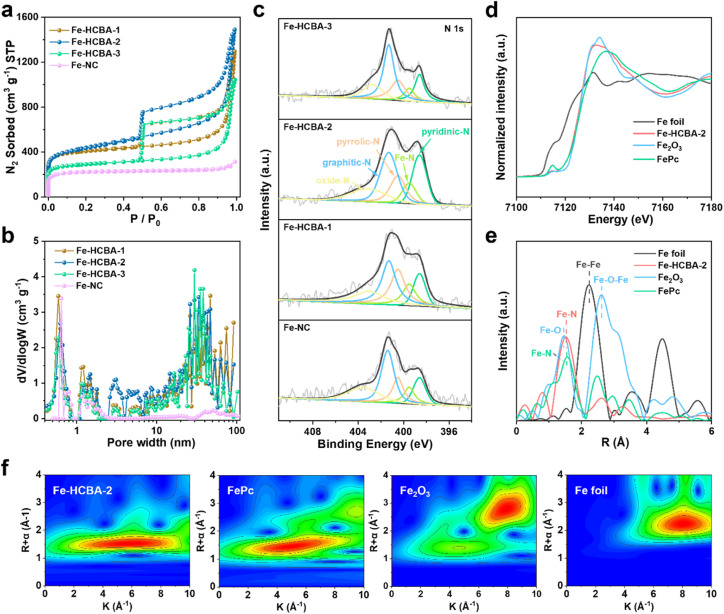
(a) N_2_ sorption isotherms, (b) NLDFT pore size distribution plots, and (c) N 1s XPS spectra of Fe-HCBA-1, Fe-HCBA-2, Fe-HCBA-3 and Fe-NC. (d) Fe K-edge XANES spectra, (e) FT *k*^2^-weighted Fe K-edge EXAFS spectra, and (f) 3D contour WT-EXAFS plots of Fe-HCBA-2 and reference samples.

The sample of ZIF-derived microporous N-doped carbon anchored with Fe SAs (Fe-NC) was prepared according to the detailed procedure provided in the ESI (Fig. S8).† The PXRD patterns (Fig. S9†) demonstrate that the broad peak at approximately 24° in all Fe-HCBA-*x* and Fe-NC samples corresponds to the (002) plane of graphitic carbon, while no peaks attributed to metallic Fe nanoparticles are detected. This observation indicates that the Fe species exist as atomically dispersed Fe SAs, consistent with the atomic-resolution HAADF-STEM characterization. The pore characteristics and BET surface areas of Fe-HCBA-*x* and Fe-NC were analyzed through N_2_ sorption measurements, with detailed information provided in Table S1.† Fe-NC displays a typical type-I isotherm, indicating its micropore-dominated pore nature. In contrast, the isotherms for all Fe-HCBA-*x* samples show large hysteresis loops (Fig. 3a) and indicate the coexistence of micropores, mesopores, and macropores. The BET surface area of Fe-HCBA-2 is significantly higher at 1570 m^2^ g^−1^, surpassing that of Fe-NC (670 m^2^ g^−1^) by 2.3 times and exceeding those of Fe-HCBA-1 (1530 m^2^ g^−1^) and Fe-HCBA-3 (1082 m^2^ g^−1^). This variation in BET surface area is not only correlated with the micropore contents but also with the highly porous carbon in the hollow cavities. Additionally, the pore size distribution analysis based on nonlocal density functional theory (NLDFT) reveals that Fe-HCBA-*x* samples exhibit a significant abundance of mesopores and macropores in the range of 10–100 nm, while Fe-NC only shows micropores (Fig. 3b). The Fe content in the Fe-HCBA-*x* and Fe-NC samples was determined using coupled plasma optical emission spectrometry (ICP-OES) (Table S2†). Given a constant Fe content in Fe^3+^/PVP/ZIF-8-*x* precursors, the notable increase in Fe from 0.55 wt%, 0.72 wt%, to 1.06 wt% for Fe-HCBA-1, -2 and -3 is attributed to the difference in carbon mass after pyrolysis (Fig. S10†), providing further evidence of the degree of hollowness exhibited by these three Fe-HCBA-*x* samples.

X-ray photoelectron spectroscopy (XPS) measurements were conducted to further investigate the elemental composition and chemical state of the Fe-HCBA-*x* and Fe-NC catalysts. The high-resolution Fe 2p spectra (Fig. S11†) indicate that all catalysts contain two pairs of peaks for Fe^2+^ (711.0 and 723.6 eV) and Fe^3+^ (715.5 and 728.4 eV), suggesting the electron-deficient characteristics of these cationic Fe species. Meanwhile, the high-resolution N 1s spectra of all catalysts can be divided into five peaks at 398.6, 399.5, 400.5, 401.3 and 403.2 eV, which originate from pyridinic-N, Fe–N, pyrrolic-N, graphitic-N and oxidized-N, respectively (Fig. 3c). The relative atomic percentages of all catalysts (Fig. S12a and Table S3†) show a positive correlation between the nitrogen content and the enhanced degree of hollowness in Fe-HCBA-*x* samples, which can be attributed to the extent of ZIF-8's decomposition. In addition, Fe-HCBA-2 has the highest content of pyridinic-N atoms (23.60%) (Fig. S12b†), which not only serve as the active sites but also coordinate with Fe atoms to form Fe–N_*x*_ active sites, thereby improving the ORR activity of the catalyst.^37^

To precisely identify the local atomic and electronic structures of Fe species in Fe-HCBA-2, synchrotron X-ray absorption near-edge structure (XANES) and extended X-ray absorption fine structure (EXAFS) spectroscopy were performed. As revealed in the Fe K-edge XANES spectra (Fig. 3d), the absorption edge of Fe-HCBA-2 lies around that of FePc and between those of the standard Fe foil and Fe_2_O_3_, indicating that the positive charge of Fe species in Fe-HCBA-2 is between 0 and +3. The Fourier-transformed EXAFS *R*-space plot in Fig. 3e displays that only a predominant peak of Fe-HCBA-2 is located at 1.53 Å, which is in line with the Fe–N first coordination sphere. Meanwhile, there are no peaks attributed to Fe–Fe and Fe–O–Fe in Fe-HCBA-2, and the FT-EXAFS fitting results (Fig. S13 and Table S4†) reveal a Fe–N_4_ coordination environment of Fe-HCBA-2. Moreover, the EXAFS wavelet transform (WT) spectra in Fig. 3f illustrate that a maximum *k* value of 5.5 Å^−1^ in Fe-HCBA-2 is identified as Fe–N(O) path, which can be distinguished from the Fe–Fe (Fe foil, 8.1 Å^−1^) and Fe–O–Fe (Fe_2_O_3_, 7.8 Å^−1^) paths. Collectively, these findings provide compelling evidence supporting the existence of atomically dispersed Fe–N_4_ sites in Fe-HCBA-2.

### Electrocatalytic performance

**Fig. 4:**
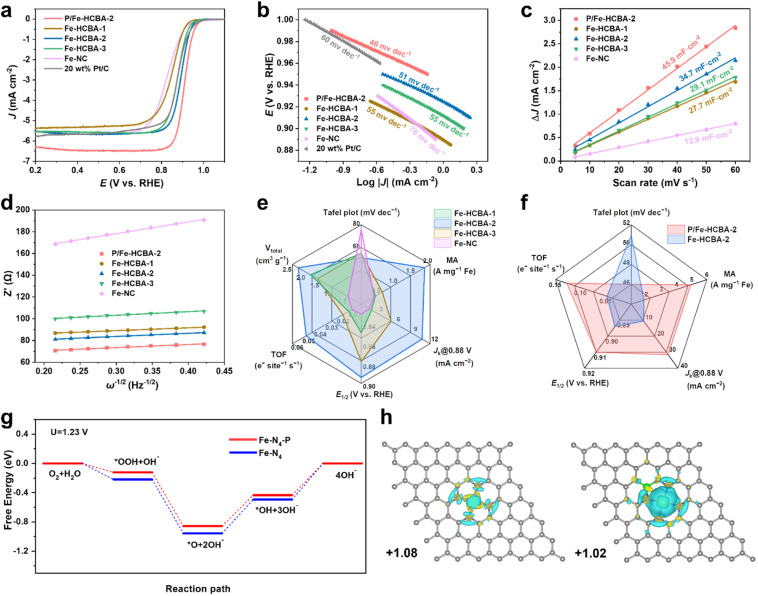
(a) LSV curves, (b) Tafel plots, (c) *C*_dl_ plots, and (d) plots of *Z*′ against *ω*^−1/2^ based on the EIS of P/Fe-HCBA-2, Fe-HCBA-1, Fe-HCBA-2, Fe-HCBA-3, Fe-NC, and commercial 20 wt% Pt/C. (e) Radar map including *J*_k_ at 0.88 V, *E*_1/2_, TOF, MA, *V*_total_, and Tafel slope of Fe-HCBA-1, Fe-HCBA-2, Fe-HCBA-3, and Fe-NC. (f) Radar map including *J*_k_ at 0.88 V, *E*_1/2_, TOF, MA, and Tafel slope of P/Fe-HCBA-2 and Fe-HCBA-2. (g) Gibbs free energy diagrams for the ORR process of the two models at *U* = 1.23 V. (h) Charge density differences and Bader charge analysis of Fe–N_4_ and Fe–N_4_–P, respectively (cyan and purple iso-surfaces denote accumulation and depletion of electron density, respectively, with an iso-surface value of 0.005 e Å^−3^).

Inspired by the desirable Fe–N_4_ active sites and structural advantages offered by the 2D array architecture, the electrocatalytic activity of Fe-HCBA-*x* and Fe-NC catalysts was explored. The ORR activity was first evaluated by linear sweep voltammetry (LSV) curves using a rotating disk electrode (RDE) in 0.1 M KOH at 1600 rpm. Fig. 4a indicates that Fe-HCBA-2 exhibits the most positive onset potential (*E*_onset_, 0.978 V) compared to Fe-HCBA-1, Fe-HCBA-3, Fe-NC and Pt/C catalysts. Notably, Fe-HCBA-2 shows a superior half-wave potential (*E*_1/2_) of 0.894 V, outperforming those of Fe-NC (0.830 V), Fe-HCBA-1 (0.849 V), Fe-HCBA-3 (0.878 V), and Pt/C (0.876 V). As shown in Fig. S14a,† the kinetic current density (*J*_k_) was extracted to evaluate the activity and mass transfer performance. As expected, the Fe-HCBA-2 exhibits an excellent *J*_k_ of 10.6 mA cm^−2^ at 0.88 V, which is 7.6, 5.9, 2.0, and 2.1 times higher than those of Fe-NC, Fe-HCBA-1, Fe-HCBA-3, and Pt/C, respectively. Furthermore, the enhanced reaction kinetics of Fe-HCBA-2 was verified by its Tafel slope (51 mV dec^−1^) in Fig. 4b, which is much lower than that of Fe-NC (76 mV dec^−1^). Considering that Fe-NC and Fe-HCBA-1 possess similar Fe content, it is noteworthy that despite their comparable *J*_k_ values, the Tafel slope of Fe-NC surpasses that of Fe-HCBA-1 significantly. This observation suggests a substantial hindrance in the utilization efficacy of Fe SAs active sites within the microporous structure of Fe-NC, consequently leading to sluggish kinetics during the ORR reaction. In addition, despite having a higher content of Fe–N_4_ sites and an almost entirely hollow structure, the ORR performance of Fe-HCBA-3 is inferior to that of Fe-HCBA-2. This observation highlights the crucial role played by the highly porous carbon frames within the hollow nanoboxes, which can significantly enlarge the BET surface area and pore volume, thereby facilitating the exposure of Fe–N_4_ active sites to reactants. The electrochemical active specific surface area (ECSA) serves as a credible metric for evaluating the surface properties of catalysts in an electrolyte, which can be deduced through the computation of double-layer capacitance (*C*_dl_) values (Fig. S15†). As depicted in Fig. 4c, the calculated *C*_dl_ of Fe-HCBA-2 (34.7 mF cm^−2^) is 2.7 times higher than that of Fe-NC (12.9 mF cm^−2^), aligning well with the observed enlargement of physical BET surface area by a factor of 2.3. This result highlights the superior ECSA and enhanced accessibility of catalytic active sites for Fe-HCBA-2.

Electrochemical impedance spectroscopy (EIS) tests were employed to investigate electron transfer and interfacial kinetics properties (Fig. S14b†). Impressively, Fe-HCBA-2 shows a significantly lower charge transfer resistance (*R*_ct_, 67.8 Ω) than that of Fe-NC (128.1 Ω), indicating that the facilitated electron transport efficiency in 2D array architecture can be attributed to its well-arranged particle configuration. In constract, the random stacking configuration of Fe-NC results in higher impedance for electron transfer.^38^ Furthermore, the boosted macroscopic reactant diffusion performance is evidenced by the quantified diffusion coefficients of hydroxide ion (*D*_OH^−^_) in the plot of *Z*′ against *ω*^−1/2^.^36,38^ As shown in Fig. 4d, the calculated *D*_OH^−^_ of Fe-HCBA-2 (1.67 × 10^−14^ cm^2^ S^−1^) is ten times higher than that of Fe-NC (1.21 × 10^−15^ cm^2^ S^−1^), while exhibiting similar values to other 2D array catalysts such as Fe-HCBA-1 (2.06 × 10^−14^ cm^2^ S^−1^) and Fe-HCBA-3 (1.25 × 10^−14^ cm^2^ S^−1^). This significant enhancement in hydroxide ion transfer efficiency highlights the indispensable role played by the 2D array architectures in improving electrocatalytic performance. To garner a more profound understanding of the utilization of active sites, the mass activity (MA) and turnover frequency (TOF) are derived by normalizing the kinetic current density at 0.88 V with respect to the corresponding Fe quantity (Fig. S14c†). As summarized in Fig. 4e, Fe-HCBA-2 presents a significantly enhanced value in MA of 1.84 A cm^−2^ and TOF of 0.048 e^−^ site^−1^ s^−1^, which are approximately 6-fold higher than those of Fe-NC, respectively. This highlights the effectiveness of the meticulously designed 2D monolayered interconnected hierarchically porous carbon nanobox architecture in maximizing the utilization of exposed active sites, facilitating electron and mass transfer, and enhancing O_2_ diffusion and transportation, thereby demonstrating the significant impact of structural modulation effect.

Considering that phosphorus (P) doping is one of the most effective strategies for enhancing the intrinsic activity of Fe–N_4_ moieties by modulating their electronic structure toward both ORR and OER, the P element was further incorporated into Fe-HCBA-2 to obtain P/Fe-HCBA-2.^39^ The characterization results of P/Fe-HCBA-2 are shown in Fig. S16–S18.† The 2D array architecture of hollow carbon nanoboxes remains well-preserved after phosphating treatment, demonstrating excellent structural integrity. The uniform distribution of the P element is observed throughout the carbon skeleton, indicating successful P doping. Additionally, the high-resolution P 2p spectrum of P/Fe-HCBA-2 displays three distinct peaks corresponding to the P–C (132.3 eV), P–N (133.2 eV), and P–O (134.1 eV) groups, respectively.^9^ Furthermore, the analysis of multiple N species in P/Fe-HCBA-2 reveals that apart from iron–nitrogen species (16.06%), pyridinic-N (22.79%) and graphitic-N (30.20%) are the predominant species, which are conducive to the electronic conduction and structural stability.^40^

The LSV curves in Fig. 4a show that the *E*_1/2_ and limit current density (*J*_L_) of P/Fe-HCBA-2 are 0.910 V and 6.3 mA cm^−2^, respectively, surpassing those of Fe-HCBA-2 (*E*_1/2_, 0.894 V; *J*_L_, 5.5 mA cm^−2^) and other advanced Fe–N–C catalysts (Table S5†). The radar map in Fig. 4f provides a comparative analysis of ORR performance indices for P/Fe-HCBA-2 and Fe-HCBA-2. Specifically, the *J*_k_ and Tafel slope of P/Fe-HCBA-2 are superior to those of Fe-HCBA-2. More importantly, the MA and TOF metrics of P/Fe-HCBA-2 have respectively escalated to be 4.94 A cm^−2^ and 0.128 e^−^ site^−1^ s^−1^, which are approximately three-fold higher than those of Fe-HCBA-2. These findings suggest that the incorporation of P atoms exerts a pivotal influence in modulating the coordination environment of the Fe–N_4_ active sites, and thus enhancing the intrinsic activity of the catalyst.

DFT calculations were performed to further explore the influence of electronic modulation in the process of ORR. According to experimental measurements, the reasonable structure of a P-doped catalyst (Fe–N_4_–P) should consist of 4 N atoms coordinating with 1 Fe atoms on the graphene sheet, and the P atom is coordinated with N and C atoms in the second shell (Fig. S19a†). For comparison, the Fe–N_4_ model was also considered (Fig. S19b†). To unveil the origin of the observed enhancement in catalytic activity and accelerated reaction kinetics of ORR for Fe–N_4_–P, Gibbs free energy diagrams of ORR were investigated. As presented in Fig. 4g, at *U* = 1.23 V, the desorption process of *OH is recognized to be the rate-determining step.^41^ Compared to that on Fe–N_4_, the overpotential of ORR on Fe–N_4_–P was lower by 0.06 V. To further probe the influence of coordination microenvironment of Fe–N_4_–P on the ORR process, the electronic structures of comparative models were investigated by calculating the difference charge densities and Bader charges of the metal active centers (Fig. 4h). Charges of 1.02 e^−^ are transferred from the Fe atom to graphene over Fe–N_4_–P, lower than that of Fe–N_4_ (1.08 e^−^), which verifies that the P atoms can promote electron accumulation in Fe sites. Furthermore, it is noted that the P doping is favorable to the electron donation from P atoms to the Fe sites, promoting the electron density in the Fe centers and thus enhancing intermediate absorption over the Fe sites. The DFT calculation results, in conjunction with the experimental findings, provide compelling evidence that P doping significantly enhances the catalytic performance of monatomic Fe.

To gain deeper insights into the perfect retention of the monatomic Fe–N_4_ active sites in P/Fe-HCBA-2, a KSCN poisoning experiment was performed. Upon the injection of KSCN solution, P/Fe-HCBA-2 displays a remarkable negative shift of 43 mV in *E*_1/2_ and a decrease in *J*_L_ to 3.7 mA cm^−2^, proving compelling evidence for the primary contribution of these monatomic Fe–N_4_ sites (Fig. S20†). Then, the rotating ring disk electrode (RRDE) measurements were carried out to determine the ORR pathways. Particularly, the peroxide yield of P/Fe-HCBA-2 is below 1% over the potential range of 0–0.8 V (Fig. S14d†), and its electron-transfer number (*n*) can be calculated as 3.99, indicating an excellent 4e^−^ selectivity during the ORR process (Fig. S21†). Fig. S22† presents the current retention situation derived from the chronoamperometric measurements to evaluate the electrocatalytic stability. Compared to Pt/C (84.7%), P/Fe-HCBA-2 exhibits remarkable stability (94.7%), highlighting its potential as a highly efficient and cost-effective alternative to traditional platinum-based catalysts. Moreover, the catalyst exhibits unparalleled robustness, as evidenced by the absence of discernible morphological or structural changes following the rigorous durability test (Fig. S23†). The remarkable methanol tolerance ability of P/Fe-HCBA-2 was further substantiated by the absence of a significant decline in current density after methanol addition (Fig. S24†).

Subsequently, the electrocatalytic OER activities of the catalysts were investigated in 1.0 M KOH electrolyte solutions. The LSV curves indicate that P/Fe-HCBA-2 exhibits an overpotential of 360 mV at 10 mA cm^−2^ (*E*_*j*_ = 10), which is comparable to the benchmark RuO_2_ (322 mV) and much smaller than that of Fe-HCBA-2 (421 mV) (Fig. S25a†). Further exploration of OER kinetics in Fig. S25b† shows that the Tafel slope of P/Fe-HCBA-2 is determined to be 110 mV dec^−1^, which is smaller than those of RuO_2_ (130 mV dec^−1^) and Fe-HCBA-2 (163 mV dec^−1^), respectively. This highlights the significant enhancement in the sluggish OER kinetics of Fe-HCBA-2 resulting from the incorporation of phosphorus atoms. Generally, the energy gap (Δ*E*) between the *E*_1/2_ for ORR and the *E*_*j*_ = 10 for OER is adopted to estimate the bifunctional catalytic activity.^42^ As illustrated in Fig. S25c†, the P/Fe-HCBA-2 shows an exceptional Δ*E* of 0.68 V, surpassing other reported bifunctional catalysts summarized in Table S6,† thereby indicating its prominent reversible electrocatalytic performance. The comprehensive electrochemical measurements unequivocally validate that the synthesis strategy, involving the construction of a 2D array architecture coupled with pore engineering and heteroatom doping, can simultaneously optimize intrinsic activity, active site utilization efficiency, and electron/mass transfer to achieve high catalytic performance.

### Rechargeable liquid and solid ZABs

**Fig. 5:**
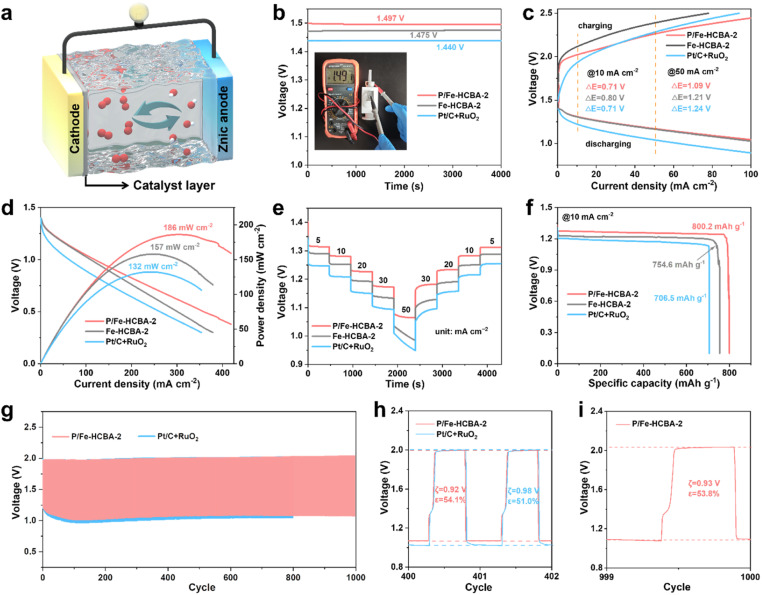
Electrochemical performance of rechargeable liquid ZABs. (a) Schematic illustration of the rechargeable liquid ZABs. (b) Open-circuit voltage. (c) Charge and discharge polarization curves. (d) Discharge polarization curves and corresponding power density plots. (e) Rate performance of different ZABs with various current densities (5, 10, 20, 30, and 50 mA cm^−2^). (f) Galvanostatic discharge curves at 10 mA cm^−2^. (g) Long-term cycling stability at 10 mA cm^−2^. (h and i) Enlarged charge–discharge curves at the 401^st^, and 1000^th^ cycle.

To evaluate the practical applicability of P/Fe-HCBA-2 in sustainable energy storage devices, we first assembled the rechargeable liquid ZABs. Fig. 5a depicts the structural schematic diagram of the liquid ZABs, featuring P/Fe-HCBA-2, Fe-HCBA-2, and Pt/C + RuO_2_ benchmark catalysts as the air cathode for comparative analysis. Consistent with the electrochemical test results, the performance of P/Fe-HCBA-2-based liquid ZABs surpasses that of Fe-HCBA-2 and Pt/C + RuO_2_ benchmark catalysts across various metrics. As illustrated in Fig. 5b–e, liquid ZABs equipped with P/Fe-HCBA-2 cathode shows the highest open-circuit potential (OCP) of 1.497 V, the lowest charge–discharge voltage gap (*ξ*) especially at relatively high current density, the largest peak power density of 186 mW cm^−2^, and optimal rate capability at different discharging current densities. In addition, the specific capacity of the rechargeable liquid ZABs was calculated by normalizing the Zn consumption at various discharging current densities. Encouragingly, the P/Fe-HCBA-2-based liquid ZABs exhibit a maximum specific capacity of 800.2 mA h g^−1^ and a corresponding highest energy density of 1024.3 W h kg^−1^ at 10 mA cm^−2^ (Fig. 5f), surpassing the performance of Pt/C + RuO_2_ cathode even at 25 mA cm^−2^ (Fig. S26†). Additionally, durability tests were conducted to assess battery output performance through charge–discharge cycles at constant current (Fig. 5g–i). Remarkably, the P/Fe-HCBA-2-based ZABs show a lower *ξ* of 0.92 V and higher energy utilization efficiency (*ε*) of 54.1% compared to the benchmark Pt/C + RuO_2_ catalyst for the 401^st^ cycle; furthermore, a negligible decrease of *ξ* and *ε* is observed even at the 1000^th^ cycle. Continuous cycling tests at varying current densities were also conducted (Fig. S27†), revealing excellent durability and reversibility of this rechargeable liquid ZABs.

**Fig. 6:**
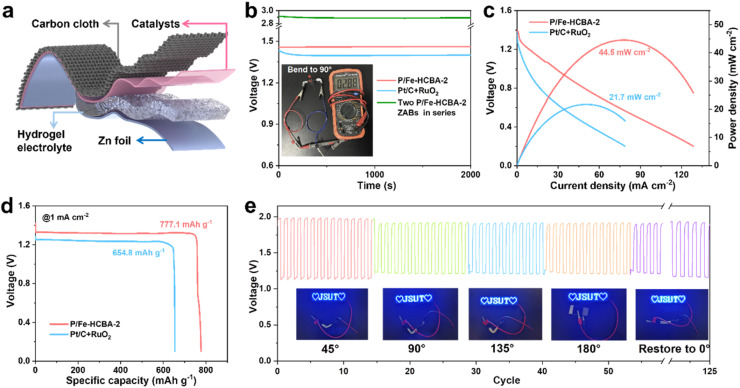
Performance evaluation of rechargeable flexible ZABs. (a) Schematic illustration of the rechargeable flexible ZABs. (b) Open-circuit voltage (inset: the stable voltage provided by two P/Fe-HCBA-2-based flexible ZABs in series). (c) Discharge polarization curves and corresponding power density plots. (d) Galvanostatic discharge curves at 1 mA cm^−2^. (e) The discharge/charge cycling stability of flexible ZABs with P/Fe-HCBA-2 cathode at 1 mA cm^−2^ under different bending states.

Motivated by the demands of miniaturized and portable electronic devices, rechargeable flexible solid-state ZABs were fabricated using carbon cloth coated with P/Fe-HCBA-2 as the air cathode and the polyacrylic acid/KOH/Zn(CH_3_COO)_2_ hydrogel polymer as the electrolyte (Fig. 6a). Flexible ZABs equipped with Pt/C + RuO_2_ cathode were also assembled. As depicted in Fig. 6b, the P/Fe-HCBA-2-based flexible ZABs deliver a higher OCV of 1.46 V compared to the Pt/C + RuO_2_-based one (1.41 V). Of particular interest, at bending angles of 0°, 45°, 90°, 135°, 180° and back to 0°, its OCV values remain consistent at levels of 1.463 V, 1.475 V, 1.474 V, 1.462 V, 1.462 V, and finally reaching again at a value of approximately 1.466 V (Fig. S28†). Additionally, the practicality of the P/Fe-HCBA-2-based flexible ZABs is further validated by their integration into a series circuit. The two-battery assembly of the flexible ZABs maintains a high OCV ranging from 2.88 to 2.90 V at various bending angles (Fig. 6b and S29†), which is approximately twice that of a single flexible battery, demonstrating its capability to power a series of LEDs (Fig. S30†) and highlighting its exceptional flexibility and versatility. As shown in Fig. 6c and d, P/Fe-HCBA-2-based flexible ZABs display a peak power density (44.5 mW cm^−2^) and specific capacity (777.1 mA h g^−1^), surpassing those achieved with Pt/C + RuO_2_ cathode (21.7 mW cm^−2^ and 654.8 mA h g^−1^, respectively), as well as the most recently reported ZABs (Table S6†). To comprehensively evaluate the flexibility of the P/Fe-HCBA-2-based flexible ZABs under external stress, we conducted in-depth investigations into their cycling stability under various bending angles. The *ξ* and *ε* of the flexible ZABs are well maintained after 125 cycles at a current density of 1 mA cm^−2^ compared to the Pt/C + RuO_2_ benchmark (Fig. 6e and S31†). Notably, this flexible ZABs shows consistent charge–discharge processes for up to 150 cycles at a current density of 2 mA cm^−2^ (Fig. S32†), highlighting its immense prospects for portable electronic devices application. These results unequivocally demonstrate that the P/Fe-HCBA-2 catalyst possesses remarkable potential for the development of next-generation energy conversion devices.

## Conclusions

In summary, we have fabricated 2D Fe^3+^/PVP/ZIF-8 hybrid monolayers with well-arranged ZIF nanopolyhedra coated with PVP and Fe^3+^ species using a facial multi-component ice-templating co-assembly approach. These monolayers serve as precursors for the construction of 2D monolayered arrays of hollow carbon nanoboxes *via* a dual-outward contraction-induced hollowing mechanism. The validity of this hollowing mechanism is demonstrated by harnessing the synergistic interfacial interaction forces. The structurally and electronically dually-modulated P/Fe-HCBA-2 catalyst exhibits remarkable catalytic performance towards both ORR and OER, which can be attributed to several fundamental strengths: (i) hollow carbon nanoboxes with ultrathin carbon walls and highly porous carbon frames provide large physical and electrochemical active specific surface area that maximizes the exposure and utilization efficiency of Fe–N_4_ active sites; (ii) the unique 2D monolayered array structure promotes electron/mass transfer efficiency; (iii) P-doping can significantly enhance the intrinsic activity of Fe–N–C catalyst by modulating electronic structure of Fe–N_4_ sites, thus enhancing the reaction kinetics. This work demonstrates a universal approach to rationally design advanced 2D monolayered hollow monatomic metal–nitrogen–carbon catalysts (M–N–C), while also emphasizing the significance of optimizing utilization efficiency and intrinsic activity of active sites, as well as facilitating electron/mass transfer of catalysts to achieve superior overall electrocatalytic performance.

## Data availability

Data supporting the findings of this study are available within the article ESI.†

## Author contributions

The manuscript was written through the contributions of all authors.

## Conflicts of interest

The authors declare no competing financial interest.

## Supplementary Material

SC-015-D4SC01257G-s001
