# Supplementary material for: 2D arrays of hollow carbon nanoboxes: outward contraction-induced hollowing mechanism in Fe–N–C catalysts
Source: Chem Sci. 2024 May 17;15(26):10110–20. doi: 10.1039/d4sc01257g (PMC11220593; doi:10.1039/d4sc01257g)
Supplement: SC-015-D4SC01257G-s001 [file SC-015-D4SC01257G-s001.pdf]

Electronic Supplementary Material (ESI) for Chemical Science.
This journal is © The Royal Society of Chemistry 2024

Supporting Information

2D arrays of hollow carbon nanoboxes: outward contraction-induced hollowing mechanism in Fe−N−C catalysts

Xiaokai Song,[a] Xiaoke Wang,[a] Jiamin Wei,*,[a] Shenghua Zhou,[a] Haifeng Wang,[b] Jiali Lou,[a] Yaqi Zhang,[a] Yuhai Liu,[a] Luyao Zou,[b] Yingji Zhao,[c] Xiaoqian Wei,[c] Sameh M. Osman,[d] Xiaopeng Li,*,[b] and Yusuke Yamauchi*,[c,e,f]

[a] *Institute of Advanced Functional Materials for Energy, School of Chemistry and Chemical Engineering, Jiangsu University of Technology, Changzhou 213001, China*

[b] *State Key Laboratory for Modification of Chemical Fibers and Polymer Materials & College of Materials Science and Engineering, Donghua University, Shanghai 201620, China*

[c] *Department of Materials Process Engineering, Graduate School of Engineering, Nagoya University, Nagoya 464-8603, Japan*

[d] *Chemistry Department, College of Science, King Saud University, P.O. Box 2455, Riyadh 11451, Saudi Arabia*

[e] *Australian Institute for Bioengineering and Nanotechnology (AIBN), The University of Queensland, Brisbane, Queensland 4072, Australia*

[f] *Department of Plant & Environmental New Resources, College of Life Sciences, Kyung Hee University, 1732 Deogyeong-daero, Giheung-gu, Yongin-si, Gyeonggi-do 17104, South Korea*

1. Experimental Procedures

1.1 Chemicals and materials

2-methylimidazole (98%) and Nafion (5 wt. % in lower aliphatic alcohols and water, contains 15-20% water; Nafion 1100EW) were purchased from Energy Chemical Co., Ltd. and Sigma-Aldrich, respectively. Other reagents and solvents were purchased from Sinopharm Co. Ltd. and used without further purification. Deionized (DI) water with resistivity higher than 18 MΩ cm^{-1} was used during the experiments.

1.2 Synthesis of ZIF nanoparticle and 2D hybrid monolayered ZIF assemblies

Truncated rhombic dodecahedral ZIF-8. ZIF-8 was prepared by a reported method.[1] Typically, a 25 mL aqueous solution of $Zn(CH_3COO)_2·2H_2O$ (1.50 g, 6.8 mmol) was prepared and added into a 25 mL aqueous solution containing 2-methylimidazole (5.60 g, 68 mmol) and cetyltrimethylammonium bromide (CTAB) (5.0 mg, 0.014 mmol) with gentle stirring for 1 min. After being left undisturbed at room temperature for 2 h, the crystals were collected by centrifugation and washed with water to be used for next step. The yield of ZIF-8 was determined based on the weight of vacuum-dried sample and used for preparing a colloidal solution with desired concentration in subsequent experiments.

2D hybrid monolayered Fe^{3+}/PVP/ZIF-8 assemblies (Fe^{3+}/PVP/ZIF-8-x). Fe^{3+}/PVP/ZIF-8-x was prepared *via* a multi-component ice-templating co-assembly strategy. Briefly, 50 mg PVP was dispersed in 30 mL of ZIF-8 colloidal solution (10 mg mL^{-1}), followed by the dropwise addition of 0.2 mL $FeCl_3$ aqueous solution (10 mg mL^{-1}) with stirring. The mixture was then stirred for another 30 min to obtain a stable colloidal solution. Subsequently, the colloid solution was transferred into a disposable paper cup, which was then slowly immersed into the liquid nitrogen for 5 min to reach full freezing of solution into ice. After freeze-drying for 48 h using a freeze-dryer, Fe^{3+}/PVP/ZIF-8-1 was obtained. Similarly, Fe^{3+}/PVP/ZIF-8-2 and Fe^{3+}/PVP/ZIF-8-3 were prepared using different amounts of PVP (75 mg and 100 mg, respectively) via this multi-component ice-templating co-assembly approach.

1.3 Synthesis of porous carbon-supported Fe−N−C catalyst

Microporous N-doped carbon anchored with Fe single atoms (Fe-NC). Fe-NC was prepared by a high-temperature pyrolysis of $Fe(acac)_3$/ZIF-8 under inert atmosphere. Firstly, $Fe(acac)_3$/ZIF-8 was synthesized by a previously reported method with a slight modification.[2] Typically, 2-methylimidazole (1.31 mg, 16 mmol) was dissolved in 15 ml methanol as solution A. $Zn(NO_3)_2·6H_2O$ (1.19 g, 4 mmol) and $Fe(acac)_3$ (35 mg, 0.1 mmol) were dissolved in 30 ml methanol under ultrasound for 15 min to form a clear solution B. Then, solution B was added into solution A with vigorous stirring for 1h at room temperature. After being left undisturbed at room temperature for 12 h, the crystals were collected by centrifugation, washed with ethanol, and vacuum-dried at 60 °C for 12 h. Subsequently, a sample of 300 mg $Fe(acac)_3$/ZIF-8 was calcined in a tube furnace at 900 °C under N_2 atmosphere for 2 h with a temperature raising rate of 3 °C min^{-1}. After natural cooling to room temperature, the catalyst was harvested and designated as Fe-NC.

2D arrays of hollow carbon nanoboxes anchored with Fe single atoms (Fe-HCBA-x). Fe-HCBA-x was prepared by a high-temperature pyrolysis of Fe^{3+}/PVP/ZIF-8-x under inert atmosphere. Typically, a sample of 300 mg Fe^{3+}/PVP/ZIF-8-x (Fe^{3+}/PVP/ZIF-8-1, Fe^{3+}/PVP/ZIF-8-2 and Fe^{3+}/PVP/ZIF-8-3) was calcined in a tube furnace at 900 °C under N_2 atmosphere for 2 h with a temperature raising rate of 3 °C min^{-1}. After natural cooling to room temperature, the catalysts were harvested and designated as Fe-HCBA-1, Fe-HCBA-2, and Fe-HCBA-3, respectively.

2D P doped Fe-HCBA-2 (P/Fe-HCBA-2). P/Fe-HCBA-2 was prepared by a previously reported method with a slight modification.[3] The 50 mg of Fe-HCBA-2 sample was added into 5 mL of triphenylphosphine (2 mg mL^{-1}) methanol solution.

Then the mixture was vigorously stirred at room temperature for 24 h. The obtained powder was annealed at 800 °C under N_2 atmosphere for 2 h. The product was washed thoroughly with plenty of water and ethanol, and then dried under vacuum for 24 h, named as P/Fe-HCBA-2.

1.4 Materials characterization

The morphology and chemical composition of the samples were investigated by scanning electron microscopy (SEM, Hitachi S-3400N) and transmission electron microscopy (TEM, JEOL JEM-2100). The surface chemistry was analyzed by X-ray photoelectron spectroscopy (XPS) performed on a AXISULTRA DLD XPS System with MONO Al source and the binding energies were calibrated using the C 1s peak at 284.8 eV. The phase composition of samples was investigated by X-ray diffraction (XRD, PANalytical X' Pert Powder). All measurements were performed at room temperature and atmospheric pressure. Brunauer–Emmett–Teller (BET) surface area of all samples was measured by N_2 adsorption and desorption at 77 K using a AUTOSORB system. The samples were degassed offline at 150 °C for 12 h under vacuum before the analysis. And the pore size distribution was analyzed by NLDFT Advanced PSD. The Fe content in the catalyst was conducted on the inductively coupled plasma-optical emission spectrometry (ICP-OES, PerkinElmer Optima 8000). XAFS measurements were obtained on the Beam line BL14W1 and BL11B at the Shanghai Synchrotron Radiation Facility (SSRF).

1.5 Electrochemical measurements

The electrochemical measurements of ORR and OER performance were tested on a CHI760E electrochemical workstation in a conventional three-electrode system at room temperature. The catalyst (4 mg) was dispersed in a mixed solution of 0.5 mL of ethanol, 0.48 mL of DI water, and 0.02 mL of 5 wt% Nafion solution, and then subjected to ultrasonication.

For electrochemical ORR measurements, the electrolytes were composed of 0.1 M KOH. An Ag/AgCl (3M KCl) electrode and a carbon rod were used as reference and counter electrode, respectively. The experiments were conducted at room temperature and all of the potentials were calibrated to a reversible hydrogen electrode (RHE). Generally, a RDE electrode with a diameter of ϕ 4 mm was used as the substrate for the working electrode. An aliquot of 20 μL of the catalyst ink was loaded onto the working electrode and dried under ambient conditions with a mass loading of 0.64 mg cm^{-2}. The electrolyte was purged by a specific gas for at least 30 min before the measurements and the gas flow was maintained during the experiments. Cyclic voltammetry (CV) measurements were conducted at a scan rate of 50 mV s^{-1} and a rotation rate of 1600 rpm. The linear scan voltammetry (LSV) curves were corrected with background current which was collected in an N_2-saturated solution. The electrochemical active specific surface area (ECSA) was derived from double-layer capacitance (C_{dl}), which was measured by a series of CV scanning in a non-Faradaic potential at various scan rates of 5-60 mV s^{-1} in N_2-saturated 0.1 M KOH. The EIS test was carried out from 10^5 Hz to 10^{-2} Hz and recorded at OCP with an amplitude of 10 mV. The tolerance of the electrocatalysts to methanol poisoning was evaluated by injecting 5 mL of 3 M methanol into the electrolyte (150 mL) during the chronoamperometric measurements. For comparison, Pt/C (20 wt% platinum, JM) was conducted on the same electrochemical tests.

To obtain the electron transfer number (n) and hydrogen peroxide yield (% H_2O_2), a rotating ring disk electrode (RRDE) electrode (diameter: ϕ 4 mm) served as working electrode. The catalyst ink (12 μL) was dropped on the RRDE electrode and then dried in the air. The % H_2O_2 and n were calculated as the following equations:

$$\%H_2O_2 = 200 \times \frac{I_R/N}{I_D + I_R/N} \quad (1)$$

$$n = \frac{4}{1 + \frac{I_R}{I_D} \times N} \quad (2)$$

Where I_D and I_R are the disk and ring currents, respectively, and N is the collection efficiency (37%) of the ring electrode.

The rotating speed of RDE was controlled from 625 to 2025 rpm. The Koutecký-Levich equation is:

$$\frac{1}{j} = \frac{1}{j_L} + \frac{1}{j_K} = \frac{1}{B\omega^{1/2}} + \frac{1}{j_K} \qquad (3)$$

$$B = 0.62nFC_0(D_0)^{2/3}\, v^{-1/6} \qquad (4)$$

$$j_K = nFkC_0 \qquad (5)$$

Where j is the measured current density, j_K and j_L are the kinetic- and diffusion-limiting current densities, ω is the angular velocity of the disk ($\omega = 2\pi N$, N is the linear rotation speed), n represents the overall number of electrons transferred in oxygen reduction, F is the Faraday constant (F = 96485 C mol^{-1}), C_0 is the bulk concentration of O_2 (1.2×10^{-6} mol cm^{-3}), D_0 is the diffusion coefficient of O_2 in 0.1 M KOH electrolyte (1.9×10^{-5} cm^2 s^{-1}), v is the kinematic viscosity of the electrolyte (0.01 cm^2 s^{-1}), and k is the electron transfer rate constant.

The kinetic mass activity (J_m) for the as-papered catalysts in this work is defined as:

$$J_m = J_k/m_{catalyst} \qquad (6)$$

Where mcatalyst is the catalyst loading on the glassy carbon disc (mg cm^{-2}), the potential to determine kinetic current density (J_k) is chosen at 0.88 V.

The turnover frequency (TOF) was calculated by the following equation:[4]

$$\mathrm{TOF} = \frac{J_k}{4\omega F m_{catalyst}} \qquad (7)$$

Where J_k is the kinetic current density at 0.88 V, ω is the content of active metal (wt.%), and F is the Faraday constant (F = 96485 C mol^{-1}).

The ion diffusion coefficient is obtained according to the following equation:[5]

$$D_{OH^-} = \frac{R^2T^2}{2A^2n^4F^4C^2\sigma^2} \qquad (8)$$

where R is the gas constant (8.314 J K^{-1} mol^{-1}), T is the room temperature (298 K), A is the the surface area of the electrode used for testing (0.07 cm^2), n is the number of transferred electrons (4), F is the Faraday constant (96485 C mol^{-1}), C is the concentration of OH^- (0.1 mmol cm^{-3}). According to $Z' = R_s + R_{ct} + \sigma\omega^{-1/2}$, it can be concluded that σ is the slope of the plot of Z' .

The ECSA was determined by the following equation:[6]

$$\mathrm{ECSA} = C_{dl}/C_s \qquad (9)$$

where Cs represents the specific capacitance of total double layer (0.04 mF cm^{-2}).

For electrochemical OER measurements, the electrolytes were composed of 1.0 M KOH. An Hg/HgO electrode and a carbon rod were used as reference and counter electrode, respectively, and a catalyst coated glassy carbon electrode (GC, diameter: ϕ 4 mm) as the working electrode. All the potentials were transformed to the reversible hydrogen electrode (RHE) in the text according to the following equation:

$$E_{vs.\mathrm{RHE}} = E_{vs.Hg/HgO} + 0.098 + 0.0592pH \qquad (10)$$

LSV was performed at a scan rate of 5 mV s^{-1} and corrected by 90% *iR* compensation to compare the performance of catalysts, where i is the measured current, and R is the compensated resistance between the working and reference electrodes.

1.6 Rechargeable liquid and flexible Zinc air batteries (ZABs)

1.6.1 Liquid ZABs

Liquid ZABs were assembled in a typical two electrode configuration, in which a metallic zinc foil of purity ~99.9% and

an air electrode were used as anode and cathode, respectively. The air electrode used a carbon paper-based gas diffusion layer (TORAY-YLS 30T). The catalyst ink was drop casted onto the gas diffusion layer and dried by a hot air gun (temperature set at 80 °C). The catalyst loading was controlled to 1.0 mg cm^{-2}. The electrolyte was aqueous 6 M KOH with 0.2 M zinc acetate, which was constantly purged with pure oxygen at a rate of 40 mL min^{-1}. Prior to performance tests, the electrolyte was also purged with oxygen for 30 min in order to saturate electrolyte with oxygen. The open circuit voltage, discharging and charging polarization curves were recorded using the CHI760E electrochemical workstation. Rate performance of the assembled ZABs were evaluated by recording voltage profiles during galvanostatic discharge at various current densities from 5 to 50 mA cm^{-2}. The constant current discharge-charge cycle curves and specific capacity of the batteries were carried out at room temperature using a battery test system (Land 3001A). The ZABs were discharged for 150 second and charged for 150 second at different current densities (10 and 15 mA cm^{-2}) in each galvanostatic cycle. The specific capacity and energy density were calculated according to the following equations:

$$\text{Specific capacity (mAh}\cdot\text{g}^{-1}) = I \times t/w_{\mathrm{Zn}}$$

$$\text{Energy density (Wh}\cdot\text{kg}^{-1}) = I \times V \times t/w_{\mathrm{Zn}}$$

where I is the applied current (A), t is the serving time (s), V is the average discharge voltage (V), and w_{Zn} is the weight of zinc consumed (g).

1.6.2 Flexible ZABs

Flexible solid-state ZAB is consisted of air electrode, polyacrylic acid (PAA) film and zinc foil, which were served as air electrode, solid electrolyte and anode, respectively. The air electrode consists of a piece of nickel foam (NF) on the air-facing side as the current collector, a gas diffusion layer (GDL) in the middle, and a carbon cloth layer coated with catalyst (catalyst loading of 1.0 mg cm^{-2}) on the electrolyte-facing side. To prepare PAA gel electrolyte: 1 g of *N*, *N*-methylene diacrylamide as a cross-linker and 5 g of acrylic acid were quickly poured into 20 mL of DI water containing 18 g of KOH under continuous stirring, followed by dissolving 1.5 g of zinc acetate. After stirring for 2 h, $K_2S_2O_8$ aqueous solution (0.12 g dissolved in 20 mL of DI water) as the initiator was added to the aforementioned solution in the square mold and then shaken vigorously to form the PAA gel. The polarization curves were recorded via linear sweep voltammetry (LSV) at room temperature on a CHI 760E electrochemical working station. Both the current density and power density were normalized to the effective surface area of the air electrode. The ZABs were discharged for 150 second and charged for 150 second at different current densities (1 and 2 mA cm^{-2}) in each galvanostatic cycle.

1.7 Computational Method

In our work, DFT calculations were implemented in the Vienna ab initio simulation package (VASP).[7,8] The projector augmented wave (PAW) method was adopted to describe interactions between ions and electrons.[9] The generalized gradient approximation (GGA) in the form of Perdew, Burke, Ernzerhof (PBE) was used to describe electron exchange and correlation.[10] The plane-wave basis set along with a kinetic cutoff energy was 400 eV. The Brillouin zones were sampled with 4 × 4 × 1 Monkhorst-Pack meshes. The structures were fully relaxed until the maximum force on each atom was less than –0.02 eV/Å and 10^{-5} eV. A vacuum space of at least 15 Å was inserted along the z direction to avoid any interactions between the periodically repeated images. The van der Waals interaction was considered using the DFT-D3 scheme.

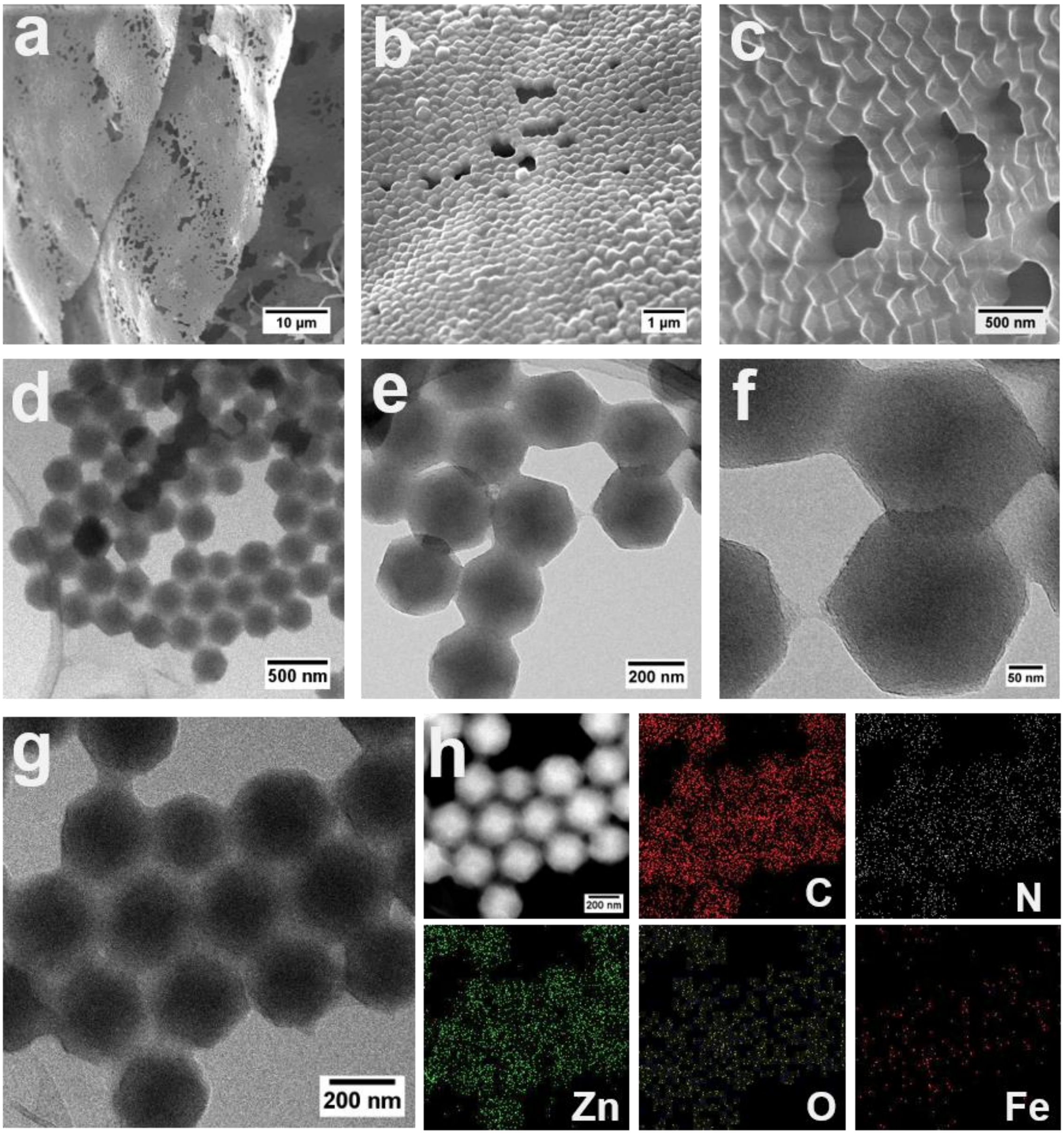

Figure S1. (a-c) SEM, (d-g) TEM, and (h) the corresponding elemental mapping of Fe^{3+}/PVP/ZIF-8-2 monolayer.

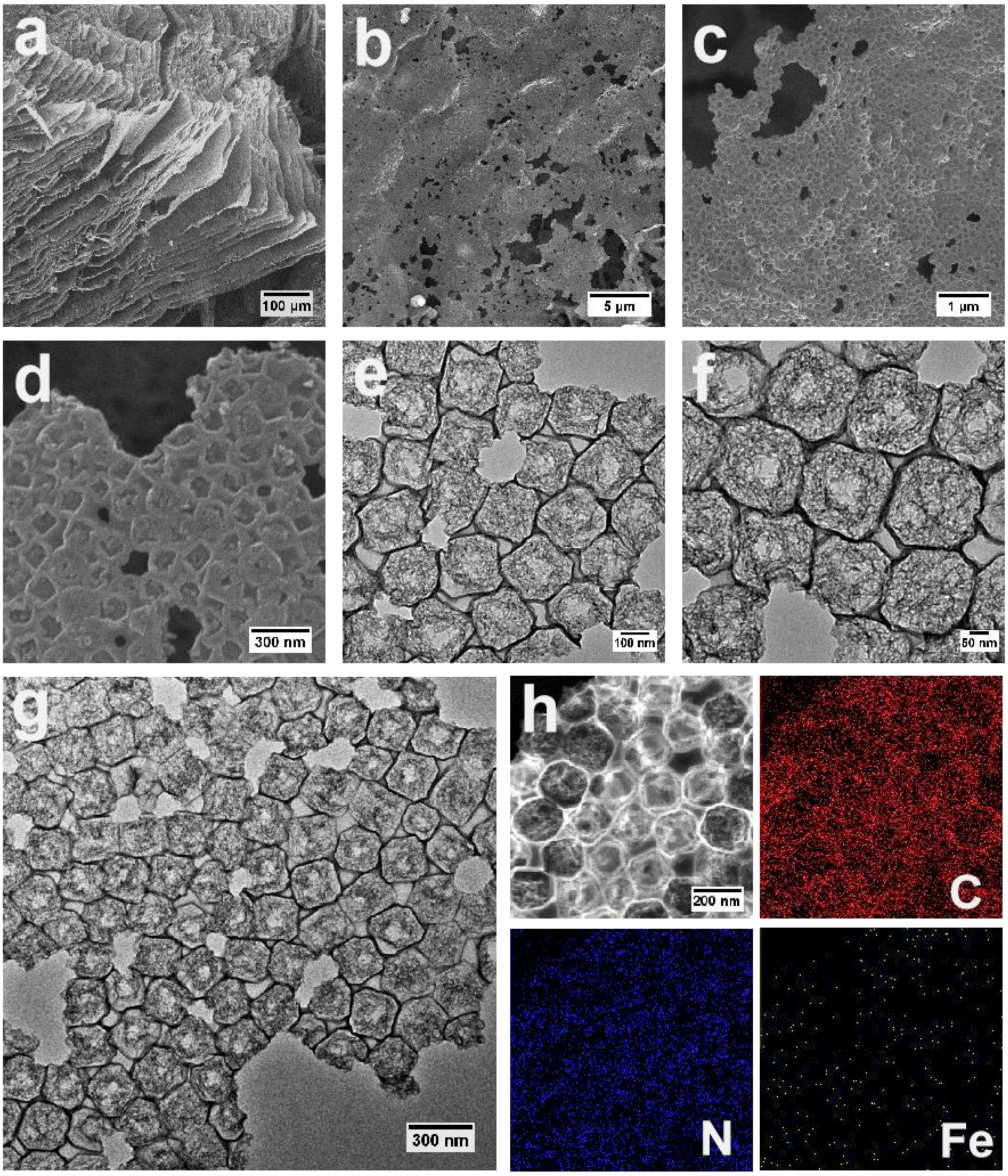

Figure S2. (a-c) SEM, (d-g) TEM, and (h) Scanning TEM (STEM) images and the corresponding elemental mapping of Fe-HCBA-2.

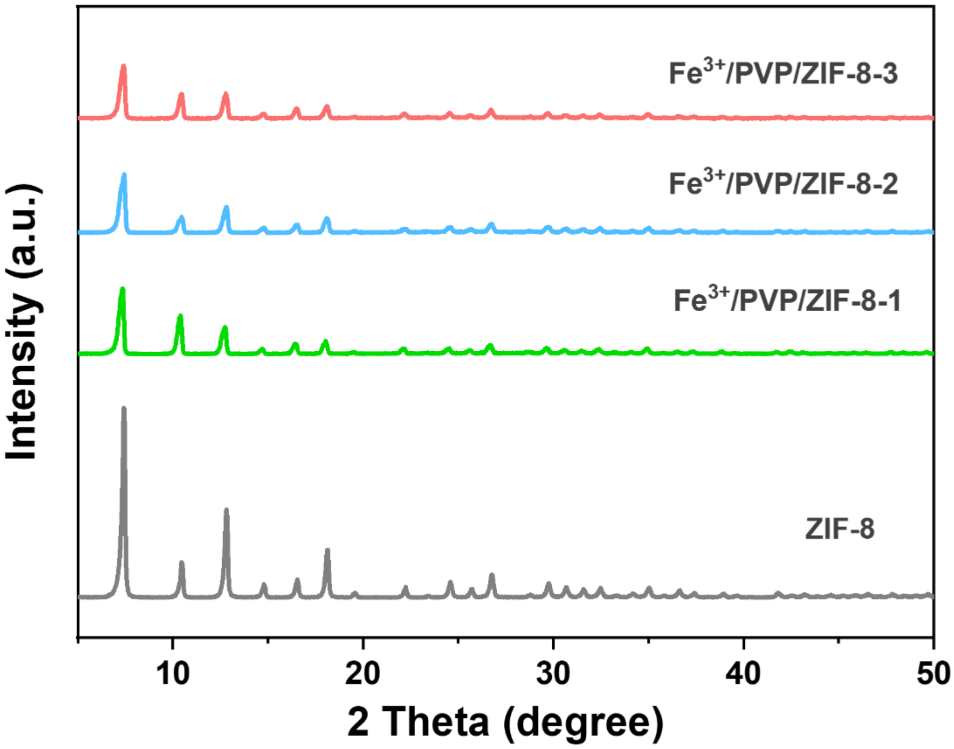

Figure S3. PXRD patterns of 2D monolayered Fe^{3+}/PVP/ZIF-8-1, Fe^{3+}/PVP/ZIF-8-2, Fe^{3+}/PVP/ZIF-8-3, and ZIF-8.

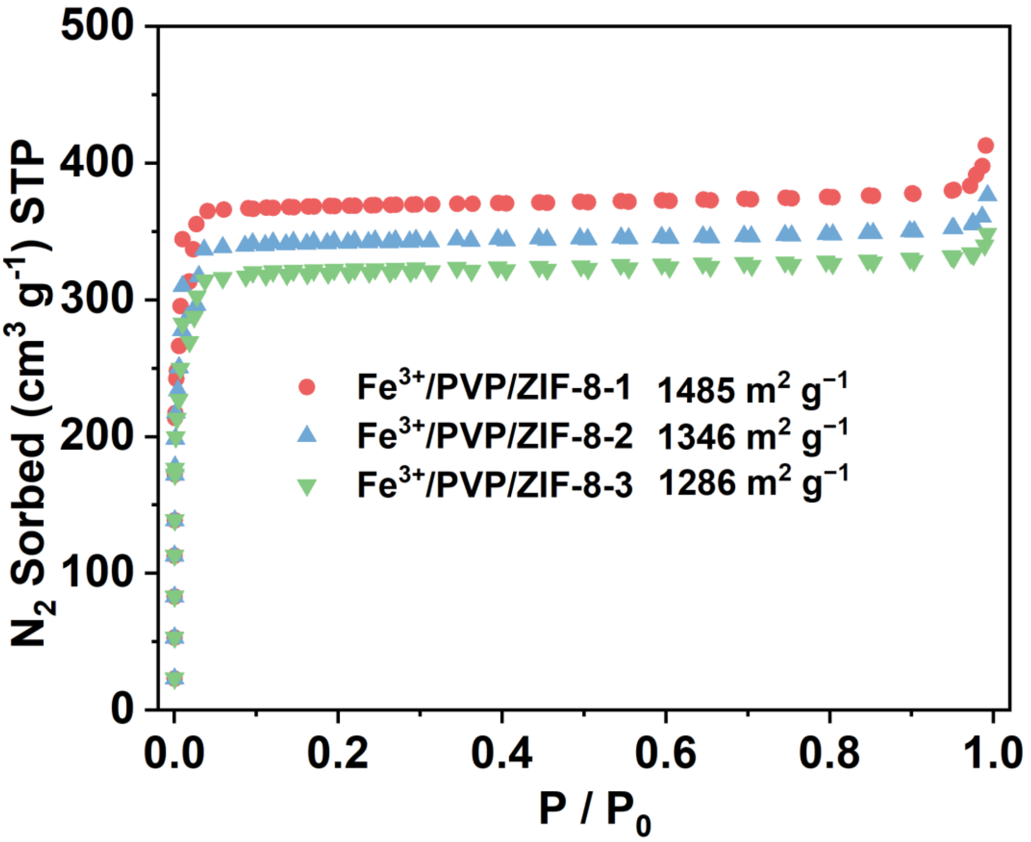

Figure S4. N_2 adsorption/desorption isotherms of 2D monolayered Fe^{3+}/PVP/ZIF-8-1, Fe^{3+}/PVP/ZIF-8-2, Fe^{3+}/PVP/ZIF-8-3.

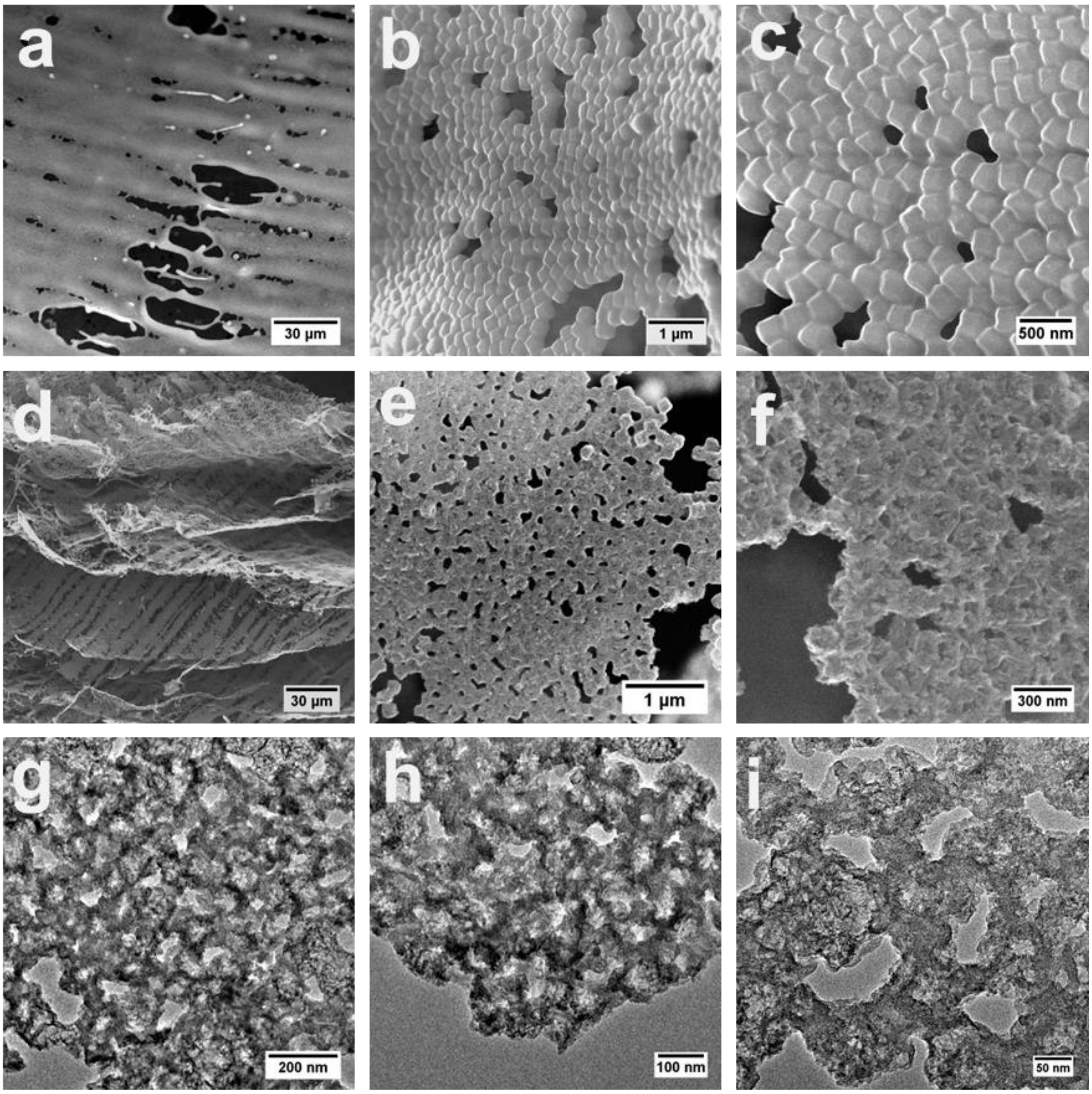

Figure S5. (a-c) SEM images of Fe^{3+}/PVP/ZIF-8-1. (d-f) SEM, and (g-i) TEM images of Fe-HCBA-1.

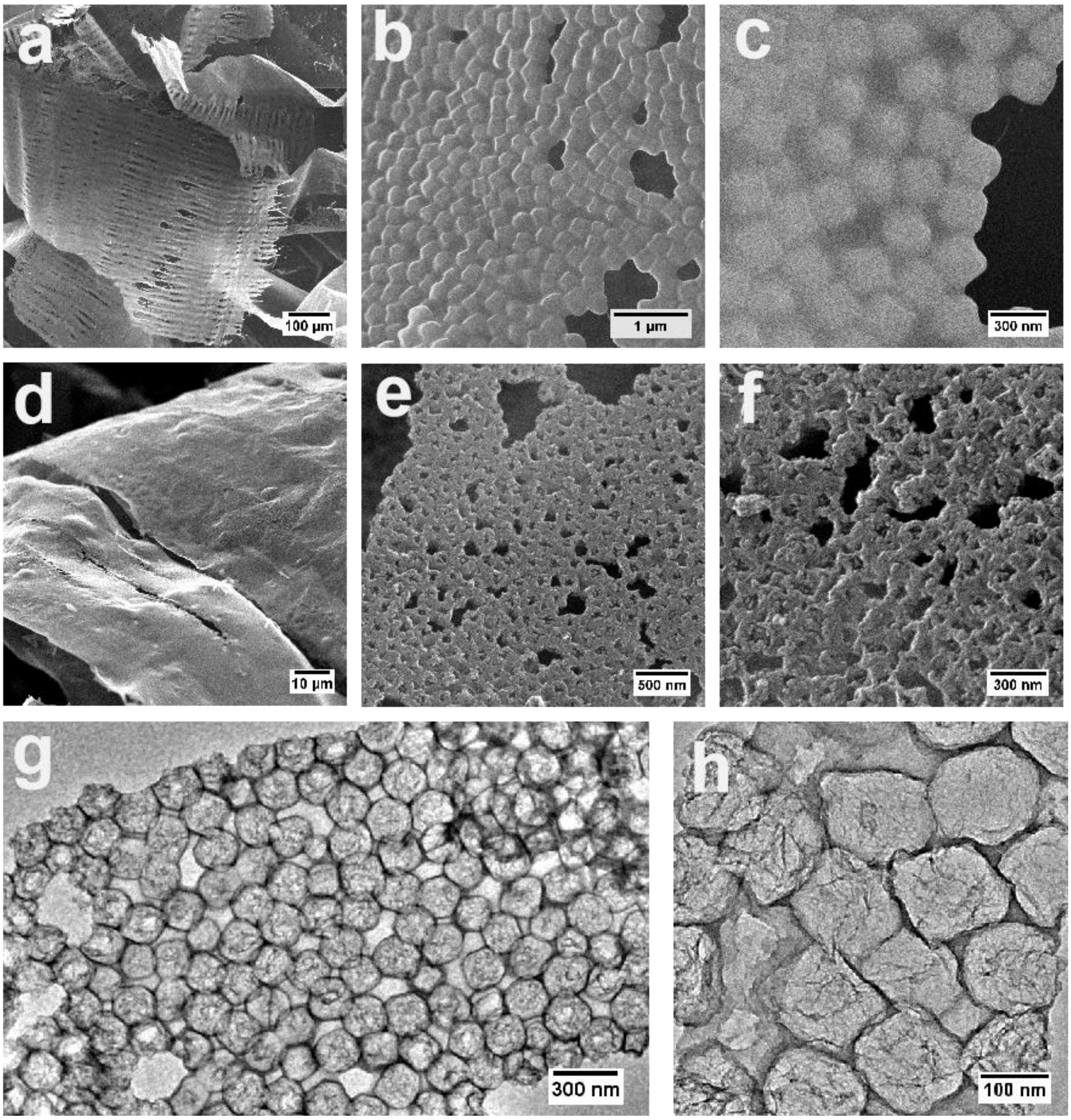

Figure S6. (a-c) SEM images of Fe^{3+}/PVP/ZIF-8-3. (d-f) SEM, and (g-h) TEM images of Fe-HCBA-3.

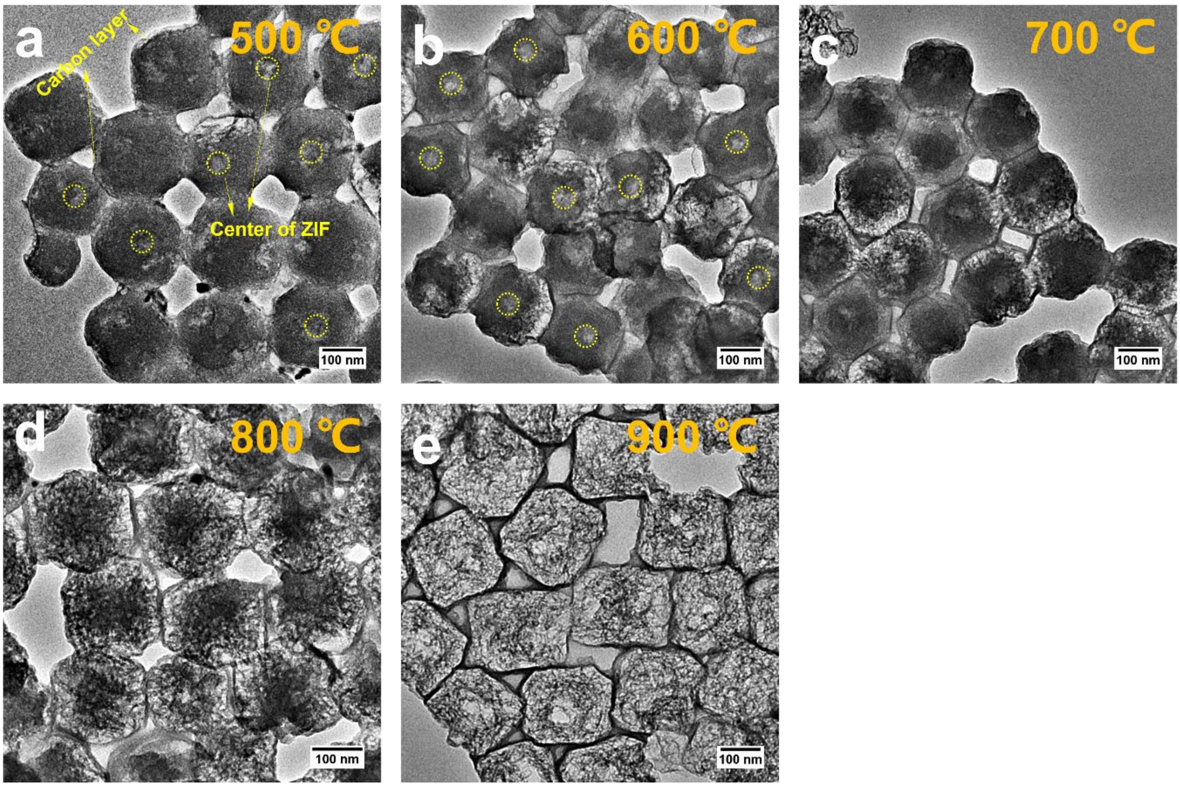

Figure S7. (a-e) TEM images of carbon at different pyrolysis temperature illustrate the temperature-dependent evolution process of carbon nanoboxes from 500 to 900 ℃.

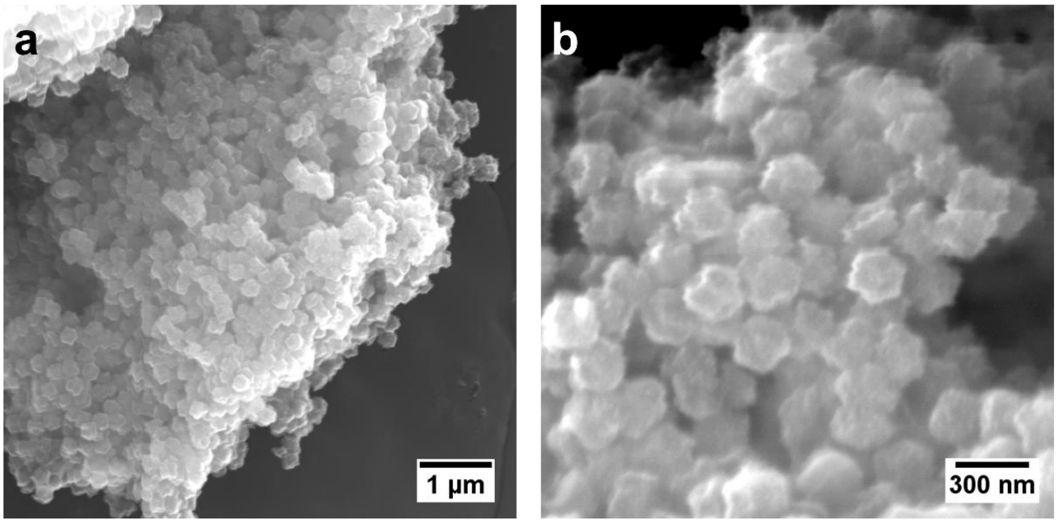

Figure S8. SEM images of Fe-NC.

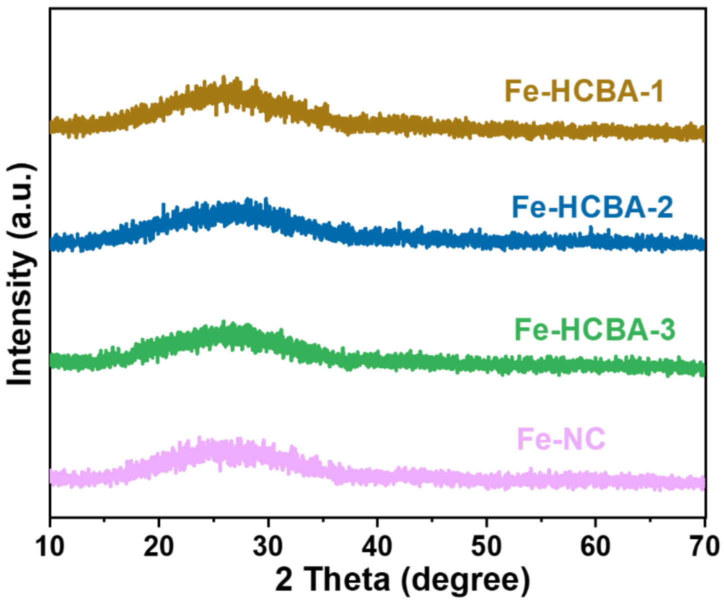

Figure S9. PXRD patterns of Fe-HCBA-1, Fe-HCBA-2, Fe-HCBA-3, and Fe-NC.

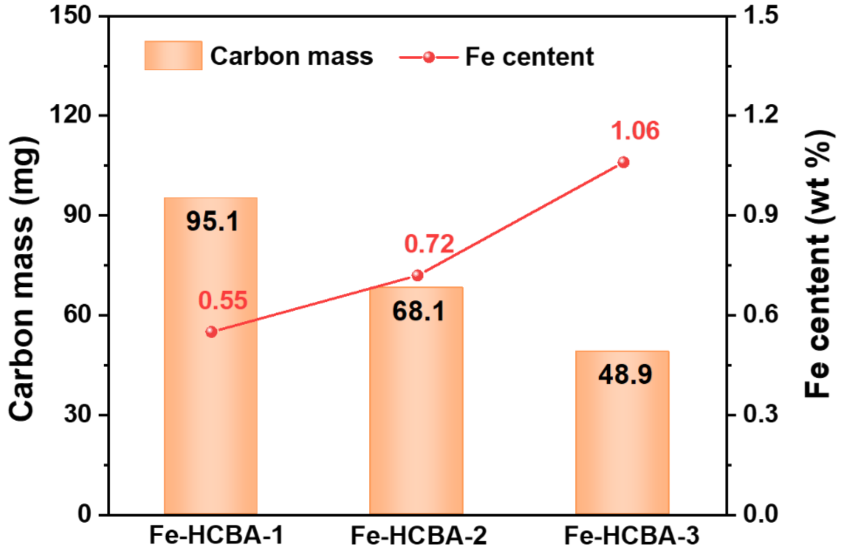

Figure S10. Relationship between Fe content and carbon mass in Fe-HCBA-1, -2 and -3 catalysts.

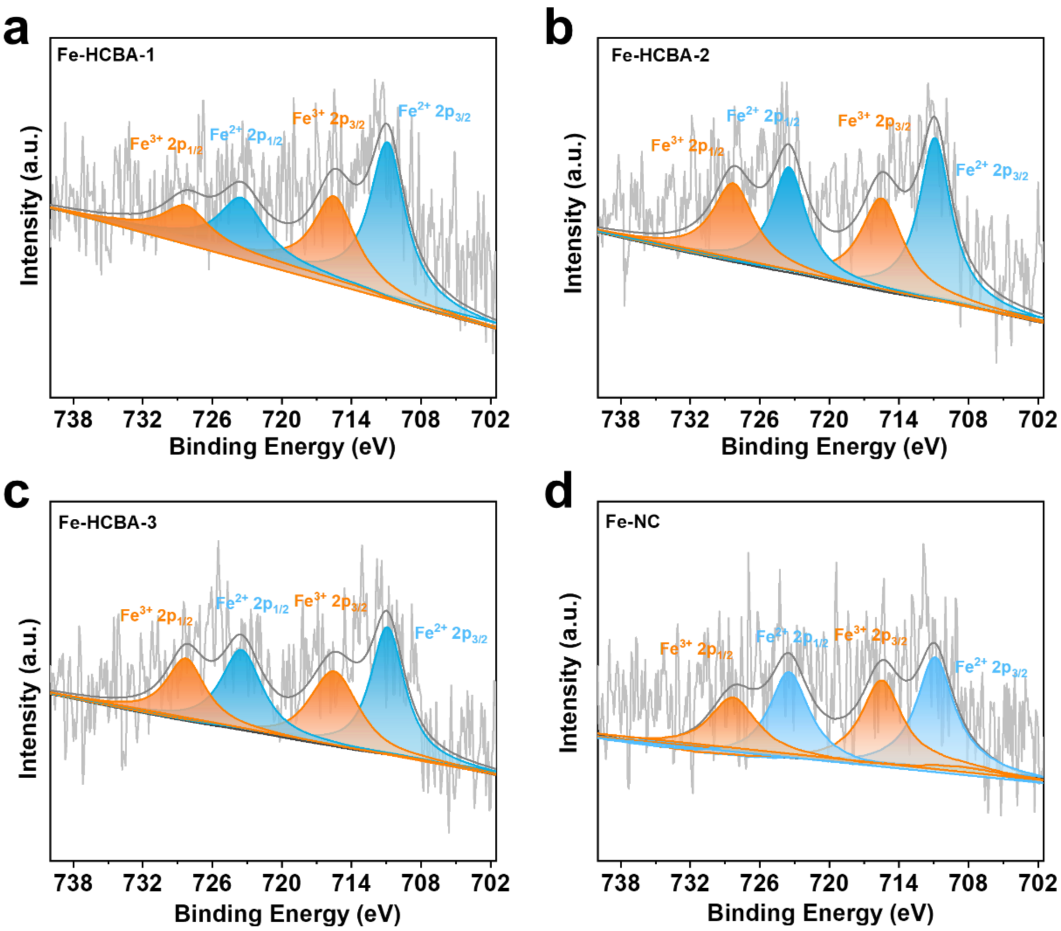

Figure S11. High-resolution Fe 2P XPS spectra of Fe-HCBA-1 (a), Fe-HCBA-2 (b), Fe-HCBA-3 (c), and Fe-NC (d).

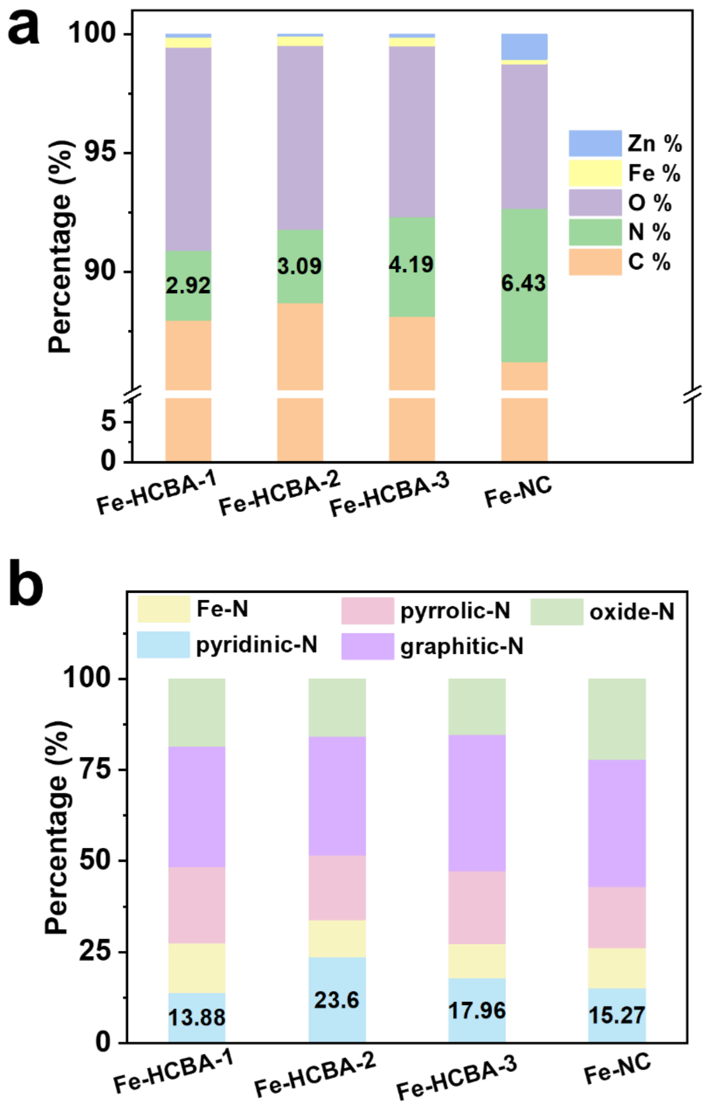

Figure S12. (a) element content, and (b) N species fraction in Fe-HCBA-1, Fe-HCBA-2, Fe-HCBA-3, and Fe-NC.

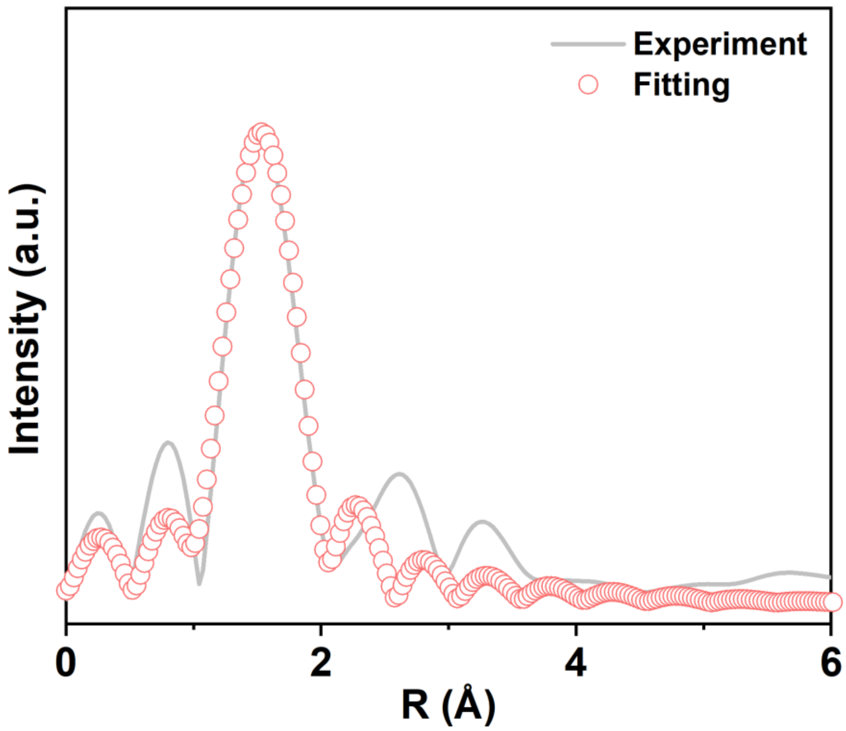

Figure S13. The EXAFS fitting curves of Fe-HCBA-2.

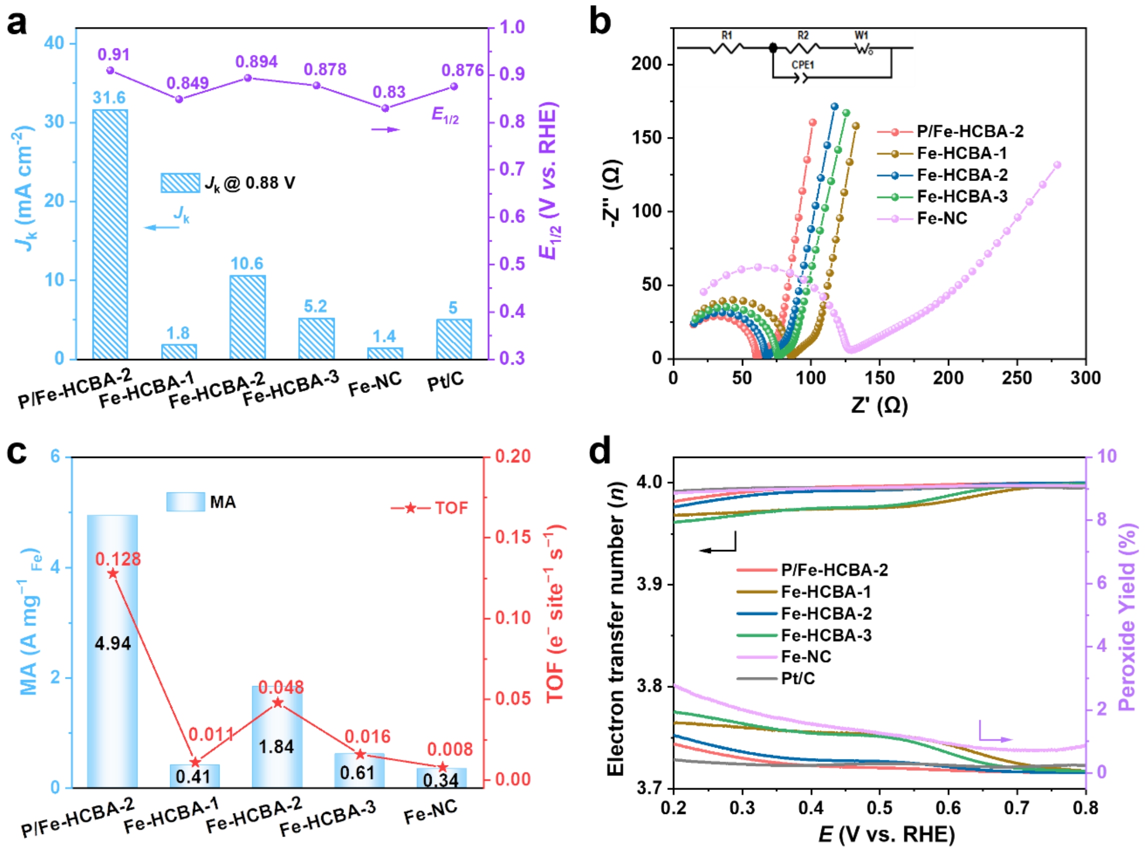

Figure S14. (a) $E_{1/2}$ and J_k at 0.88 V vs. RHE, (b) EIS spectra of catalysts recorded at open-circuit potential, (c) M_A and TOF value, and (d) H_2O_2 yield and electron-transfer number of P/Fe-HCBA-2 and counterpart catalysts.

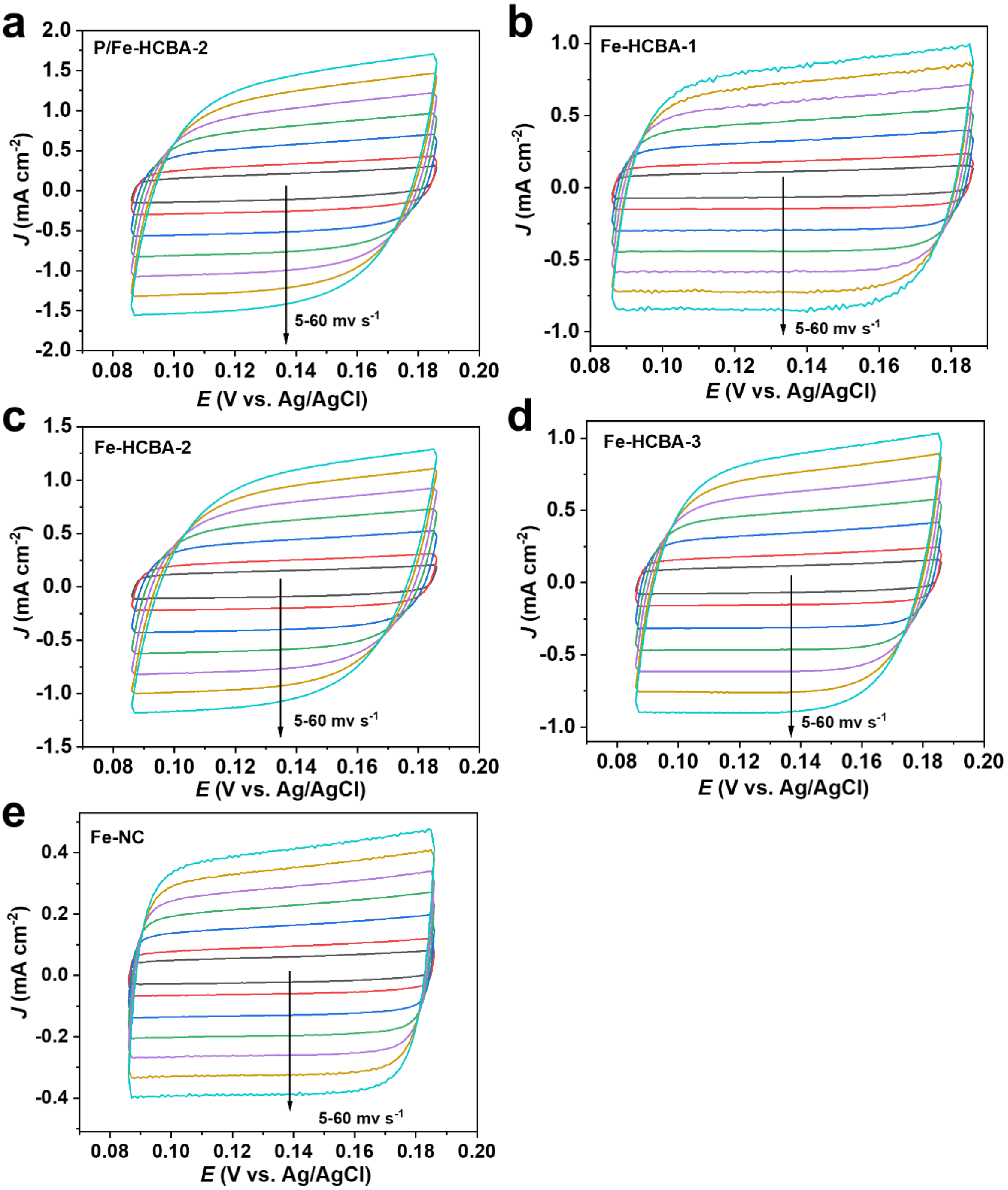

Figure S15. The cyclic voltammograms curves in the non-faradaic region at different scan rates: (a) P/Fe-HCBA-2, (b) Fe-HCBA-1, (c) Fe-HCBA-2, (d) Fe-HCBA-3, and (e) Fe-NC.

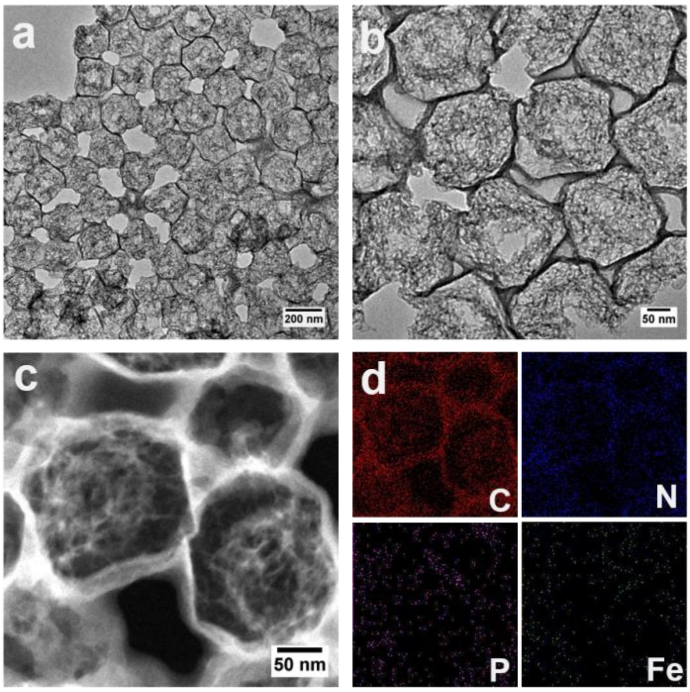

Figure S16. (a-b) TEM, (c) Scanning TEM (STEM) image, and (d) the corresponding elemental mapping of P/Fe-HCBA-2.

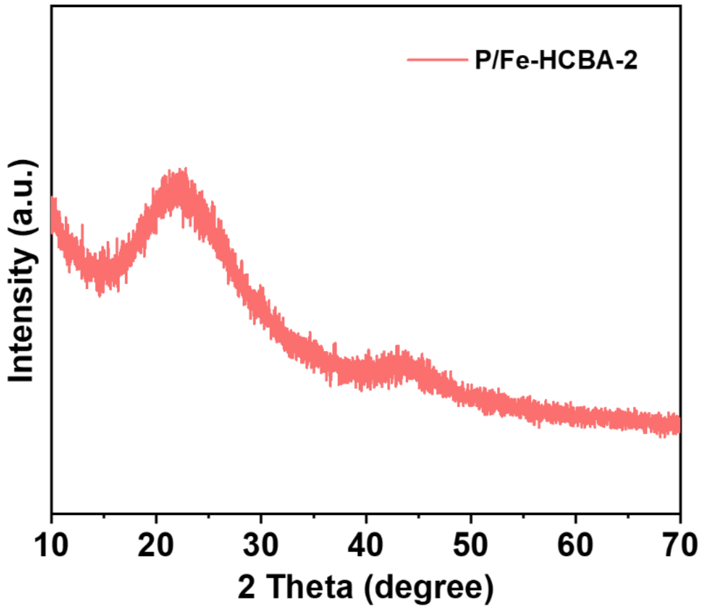

Figure S17. PXRD patterns of P/Fe-HCBA-2.

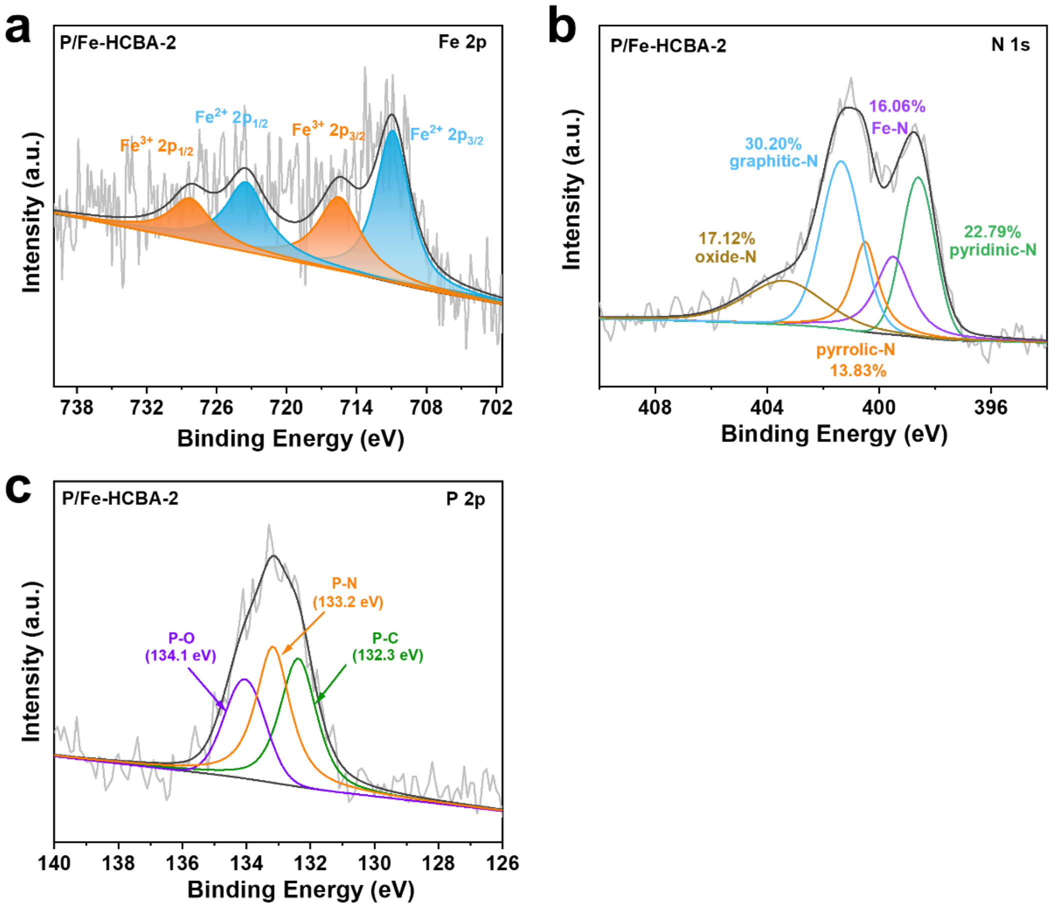

Figure S18. High-resolution (a) Fe 2P, (b) N 1s and (c) P 2p XPS spectra of P/Fe-HCBA-2.

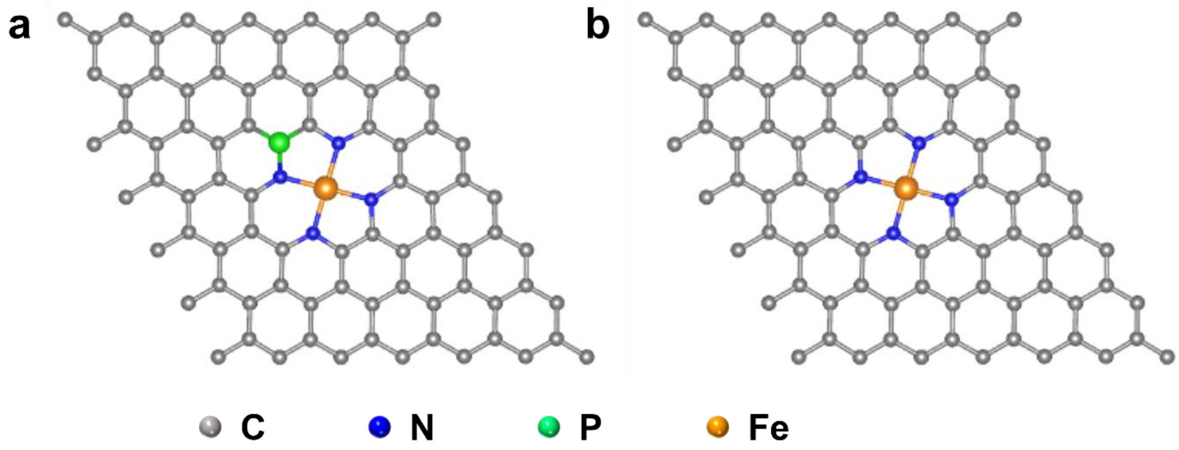

Figure S19. Structural configurations of (a) Fe–N_4–P and (b) Fe–N_4.

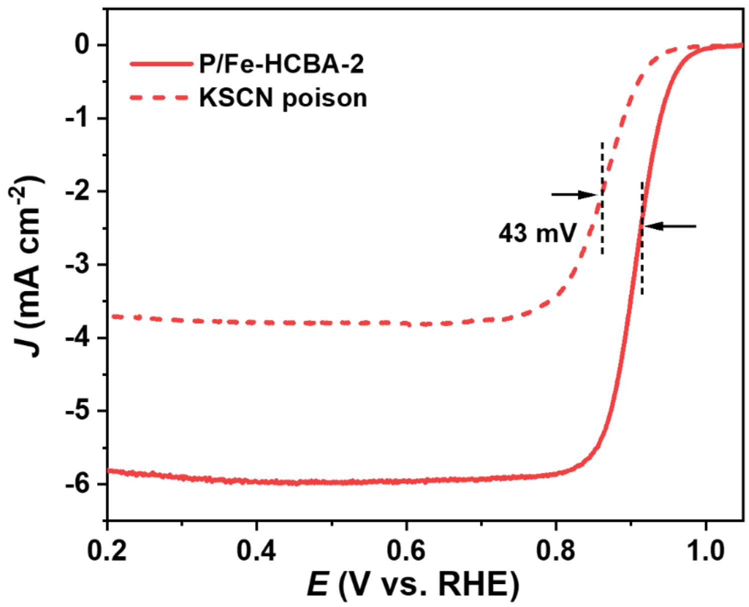

Figure S20. ORR polarization curves of P/Fe-HCBA-2 before and after KSCN injection.

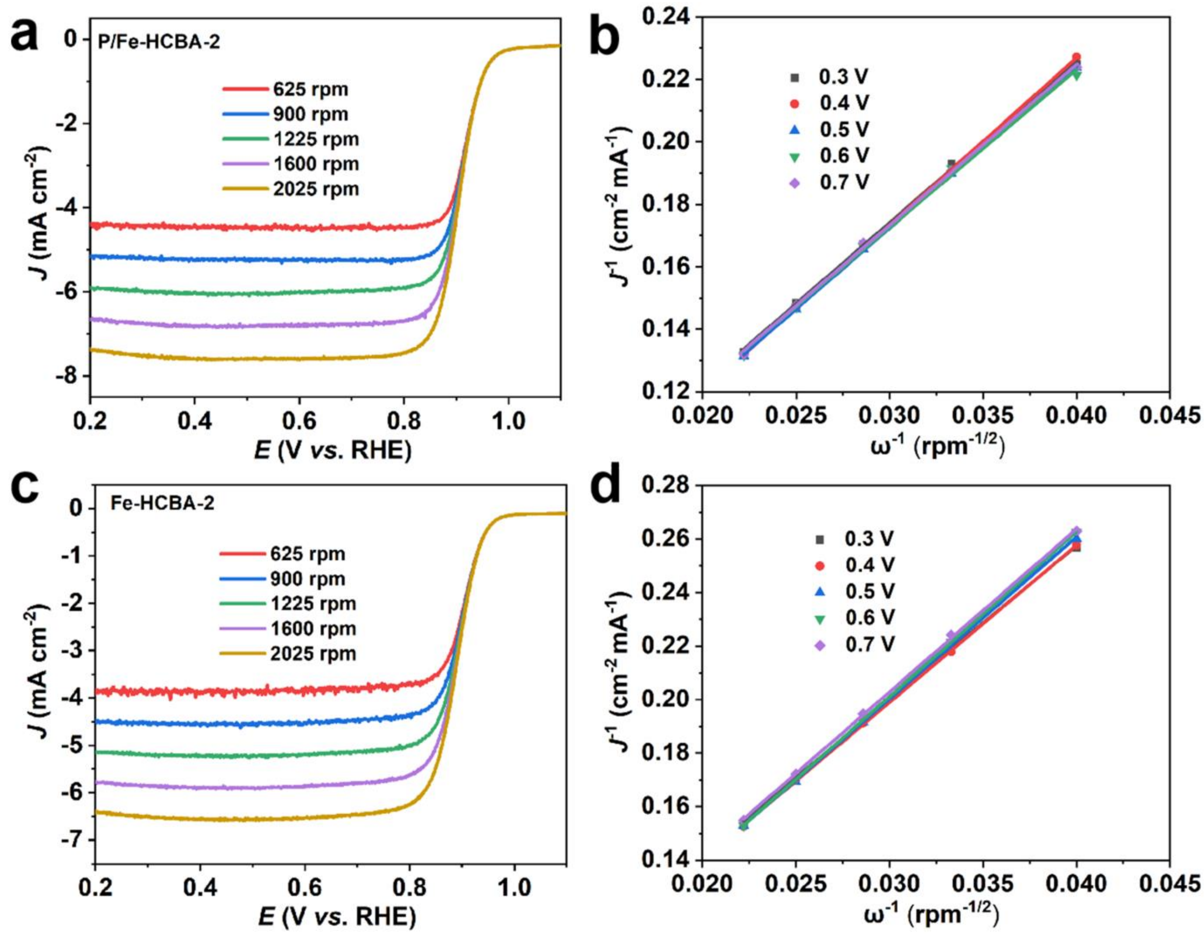

Figure S21. LSV curves at a scan rate of 2 mV s^{-1} in 0.1 M KOH with rotating speed from 625 to 2025 rpm. (a) ORR polarization curves of P/Fe-HCBA-2, and (b) corresponding K–L plots. (c) ORR polarization curves of Fe-HCBA-2, and (d) corresponding K–L plots.

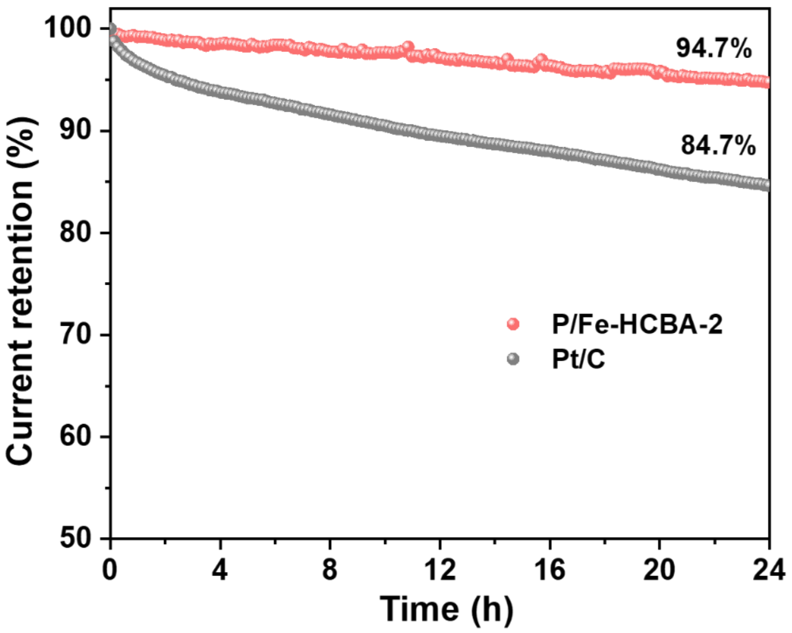

Figure S22. Current retention from chronoamperometric curves of P/Fe-HCBA-2 and 20 wt % Pt/C.

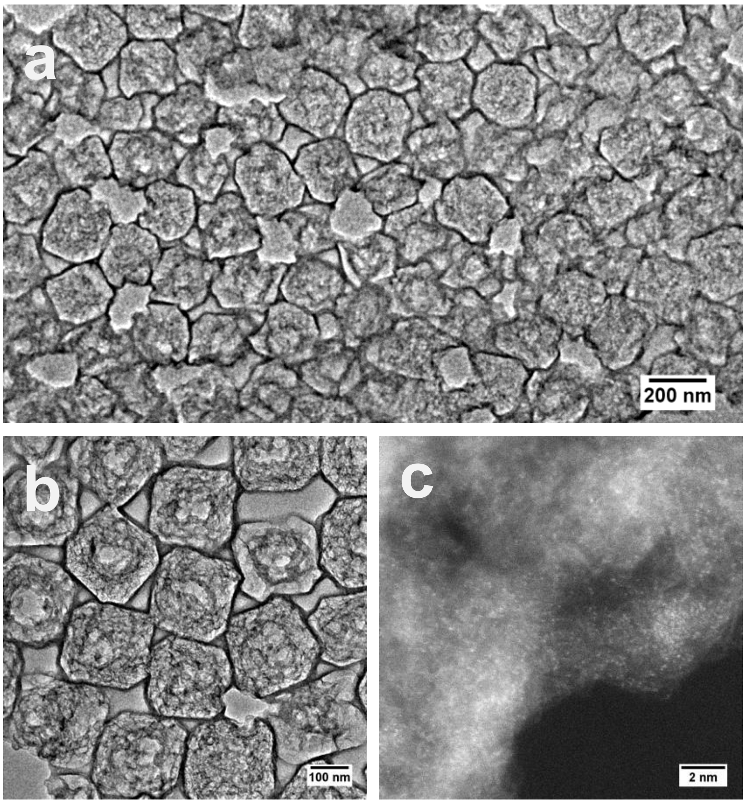

Figure S23. (a-b) TEM, and (c) Atomic-resolution HAADF-STEM of P/Fe-HCBA-2 after the stability test.

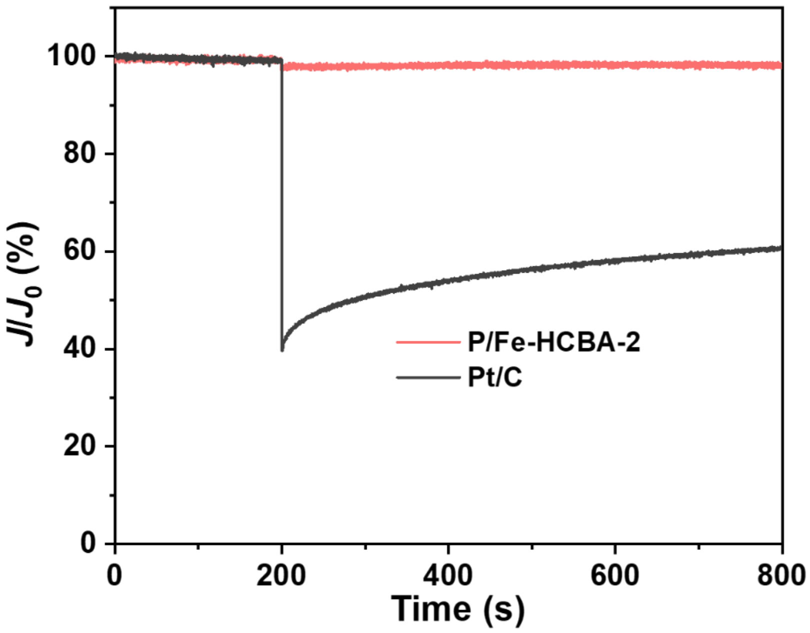

Figure S24. Chronoamperometric responses of P/Fe-HCBA-2 and Pt/C with the addition of methanol at around 200 s.

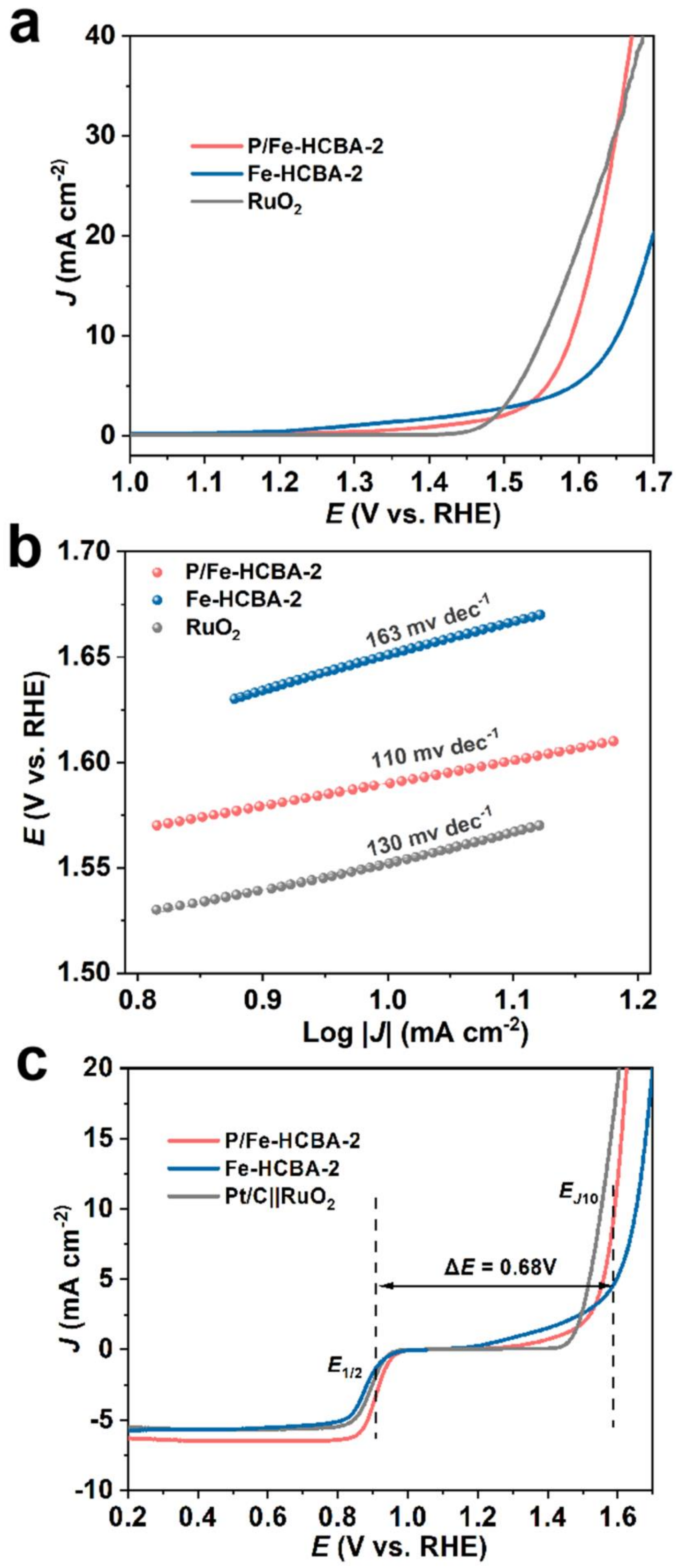

Figure S25. (a) OER polarization curves with *iR*-correction, and (b) corresponding Tafel plots of P/Fe-HCBA-2, Fe-HCBA-2, and RuO_2. (c) The overall polarization curves of different electrocatalysts.

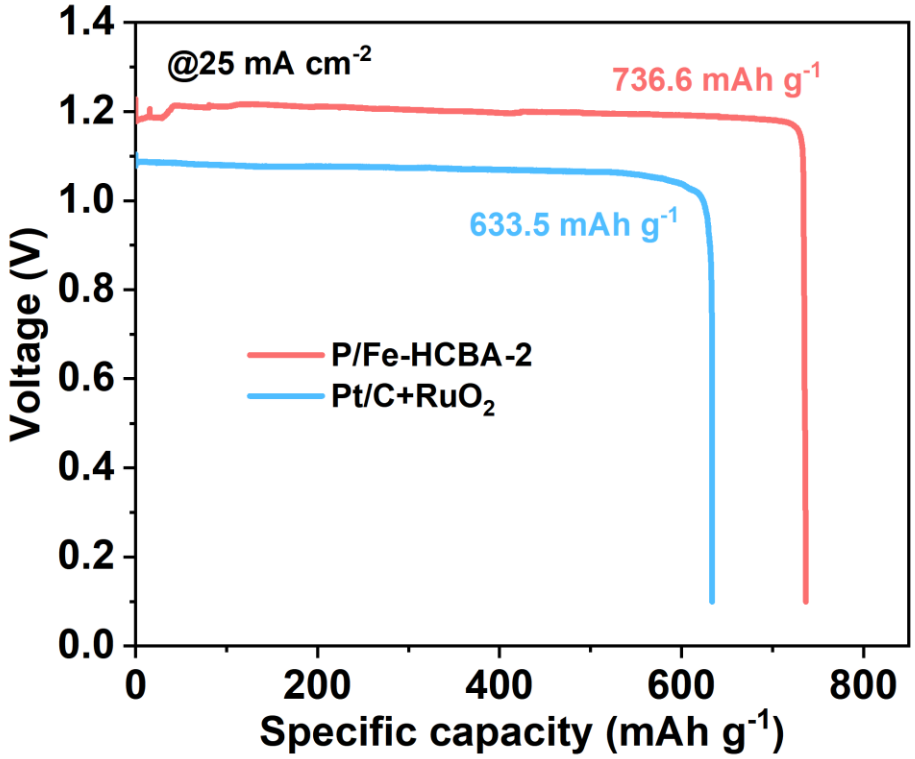

Figure S26. Galvanostatic discharge curves at 25 mA cm^{-2} for the liquid ZABs assembled with P/Fe-HCBA-2 and Pt/C+RuO_2 catalysts.

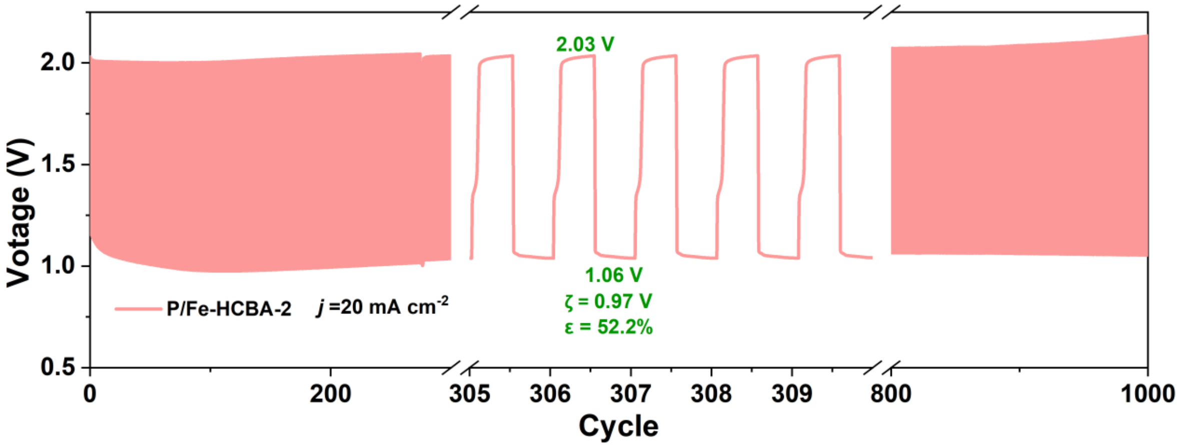

Figure S27. Galvanostatic charge–discharge curves of liquid ZABs with P/Fe-HCBA-2 cathode at 20 mA cm^{-2}.

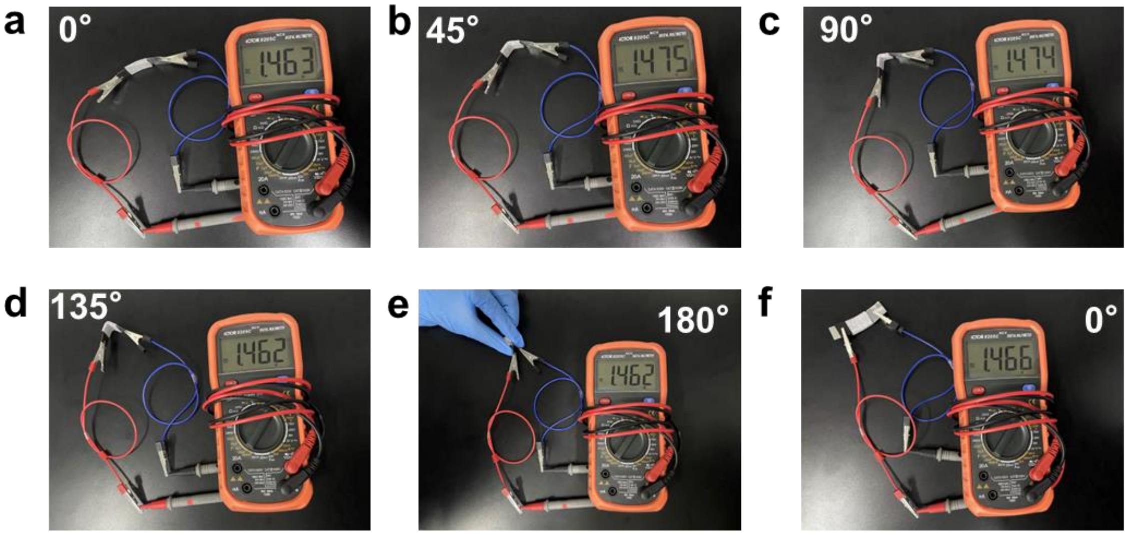

Figure S28. The open circuit voltage of the flexible ZABs with P/Fe-HCBA-2 cathode under different bending angles.

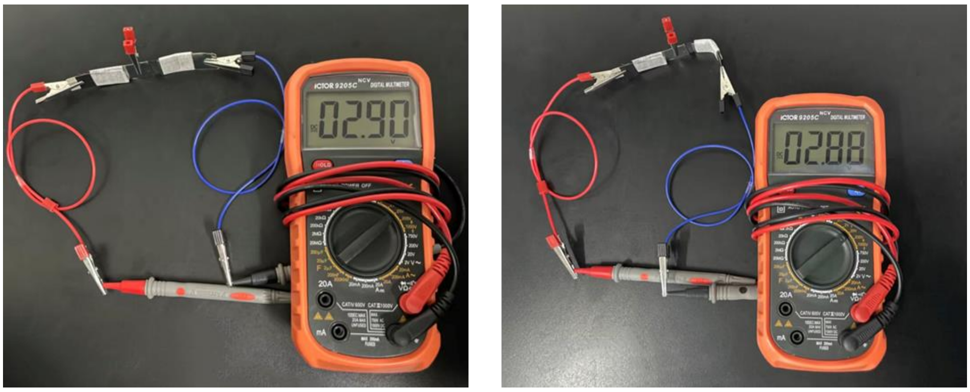

Figure S29. The open circuit voltage of two series flexible ZABs with P/Fe-HCBA-2 cathode.

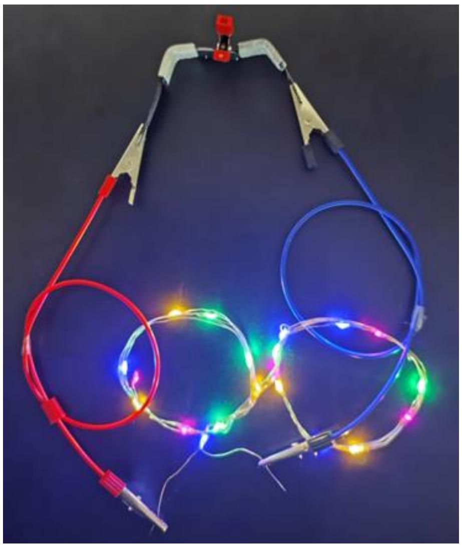

Figure 30. Digital photos of two series P/Fe-HCBA-2-based flexible ZABs powering several LED lights.

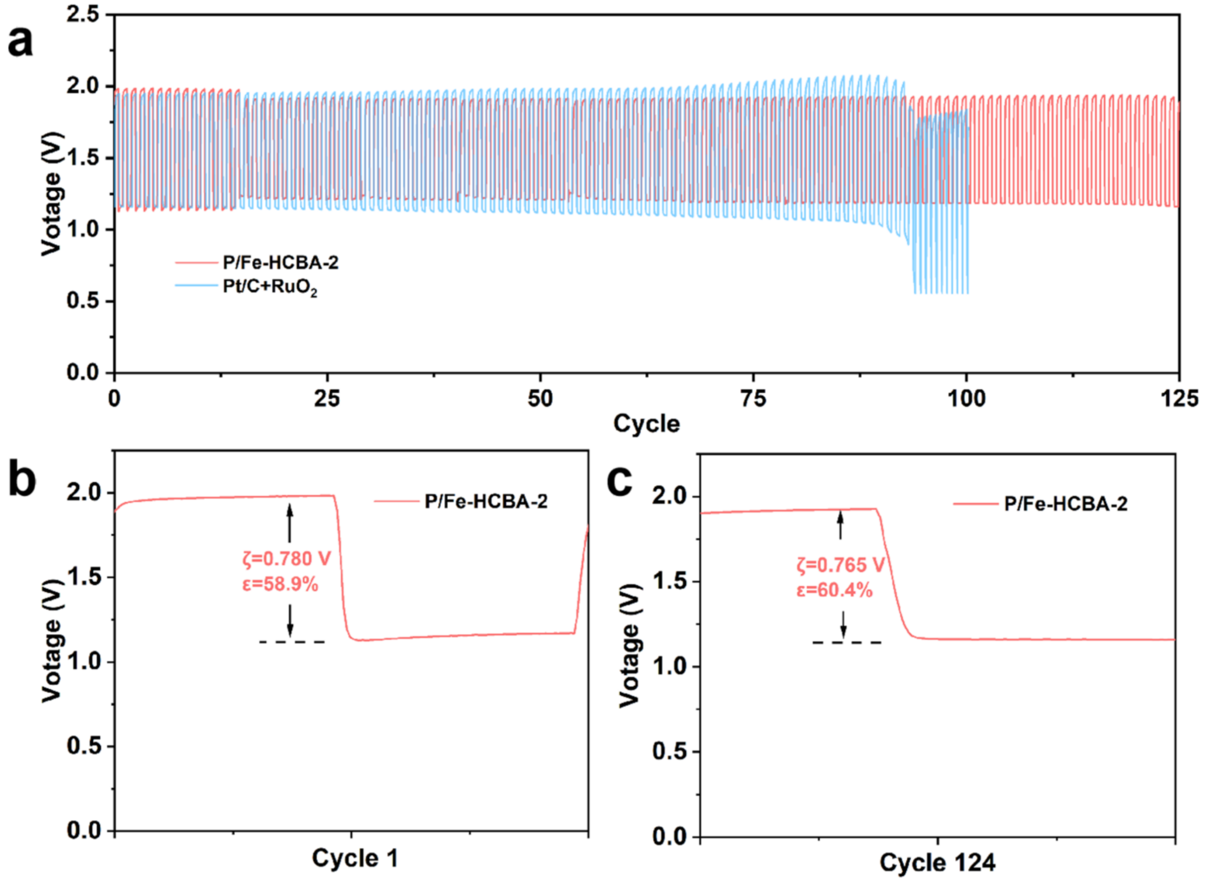

Figure S31. (a) Galvanostatic discharge/charge cycling capability of flexible ZABs with P/Fe-HCBA-2 cathode and Pt/C+RuO_2 at 1 mA cm^{-2}. (b–c) The 1st, and 124th charge–discharge cycle curves of P/Fe-HCBA-2-based flexible ZABs (The charge and discharge plateau voltages used here are their average values).

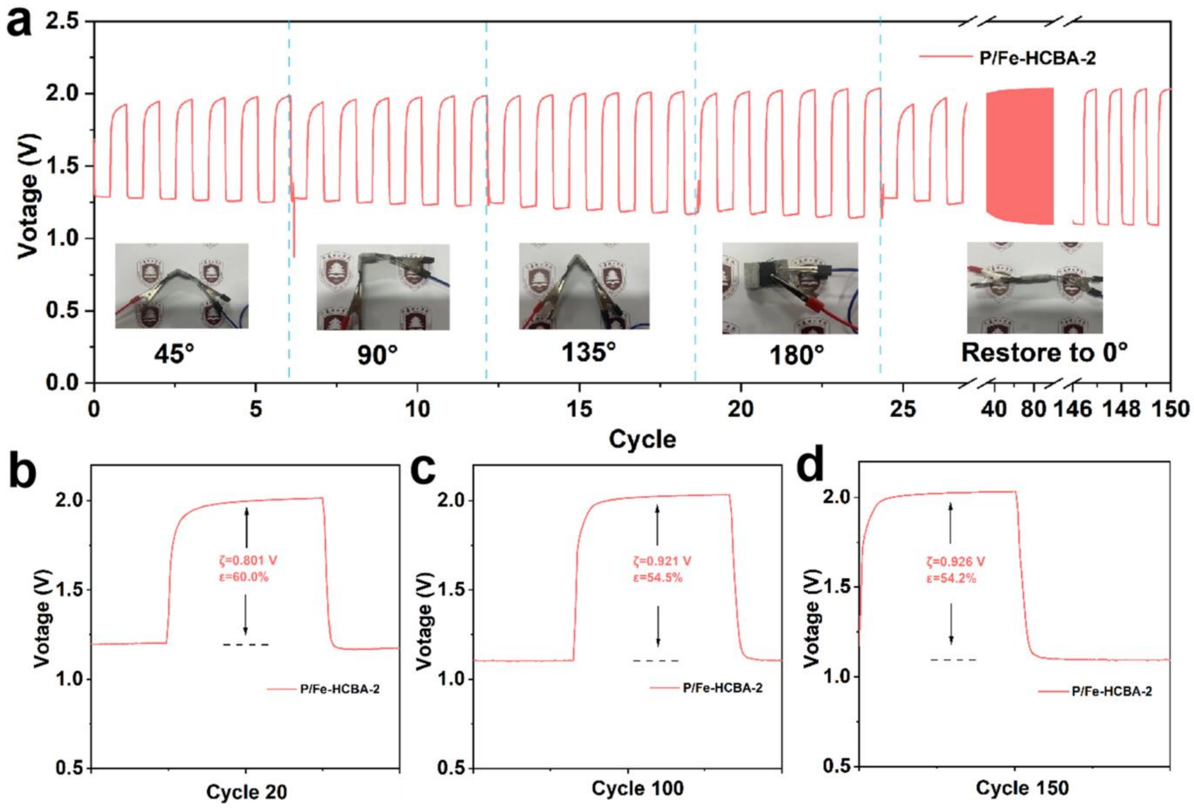

Figure S32. (a) Galvanostatic discharge/charge cycling capability of flexible ZABs with P/Fe-HCBA-2 cathode at 2 mA cm^{-2} under various folding angles. (b–d) The 20th, 100th, and 150th charge–discharge cycle curves of P/Fe-HCBA-2-based flexible ZABs.

Table S1. The BET surface area and the total pore volume of different samples.

Sample	Specific surface area S_{BET} ($m^2\ g^{-1}$)	Total pore volume V_{total} ($cm^3\ g^{-1}$)
Fe^{3+}/PVP/ZIF-8-1	1485	0.63
Fe^{3+}/PVP/ZIF-8-2	1346	0.57
Fe^{3+}/PVP/ZIF-8-3	1286	0.53
Fe-HCBA-1	1530	1.59
Fe-HCBA-2	1570	2.29
Fe-HCBA-3	1082	1.59
Fe-NC	670	0.48

Table S2. The amount of Fe in different samples determined by ICP-OES.

Samples	Fe percentage (wt%)
P/Fe-HCBA-2	0.80%
Fe-HCBA-1	0.55%
Fe-HCBA-2	0.72%
Fe-HCBA-3	1.06%
Fe-NC	0.52%

Table S3. Elemental composition of different samples determined by XPS.

Sample	P (at%)	C (at%)	N (at%)	O (at%)	Fe (at%)	Zn (at%)
P/Fe-HCBA-2	0.92	87.31	3.21	8.10	0.33	0.13
Fe-HCBA-1	/	87.98	2.92	8.56	0.43	0.11
Fe-HCBA-2	/	88.70	3.09	7.74	0.38	0.09
Fe-HCBA-3	/	88.12	4.19	7.19	0.36	0.14
Fe-NC	/	86.22	6.43	6.09	0.21	1.05

Table S4. EXAFS fitting parameters at the Fe K-edge EXAFS fitting of Fe-HCBA-2.

sample	shell	N	R (Å)	$\sigma^2(10^{-3}Å^2)$	ΔE_0 (eV)	R factor
Fe-HCBA-2	Fe-N	4.3	2.03	3.0	2.6	0.002

N, coordination numbers; R, the internal atomic distance; σ^2, the Debye-Waller factor; ΔE_0, inner potential correction to account for the difference in the inner potential between the sample and the reference compound; R factor, indicates the goodness of the of the fitting.

Table S5. Comparison of ORR catalytic activity between P/Fe-HCBA-2 and other previously reported single-atom Iron ORR electrocatalysts in 0.1 M KOH.

**Smaple**	**Onset potential (V vs. RHE)**	**Half-wave potential (V vs. RHE)**	**Tafel slope (mV dec^{-1})**	**Limit current density (mA cm^{-2})**	**Ref**
P/Fe-HCBA-2	0.996	0.91	45	6.3	this work
Fe_1Co_3-NC-1100	1.05	0.877	69.06	N/A	*ACS Catal.* **2022**, 12, 1216−1227
FePc@Co-SAs/PCNF	0.99	0.87	51	N/A	*Chem. Eng. J.* **2022**, 450, 138213
Fe SA/NCZ	1.00	0.87	70	N/A	*Adv. Funct. Mater.* **2023**, 33, 2213897
Fe-N-NDC-1-900	1.06	0.89	61	6.1	*J. Mater. Chem. A* **2021**, 9, 5556
Fe/SNCFs-NH_3	1.02	0.89	70.82	6.1	*Adv. Mater.* **2022**, 34, 2105410
Fe_3Co_7-NC	1.02	0.893	56	6.08	*Adv. Funct. Mater.* **2023**, 33, 2212299
Fe/N/C-MW	N/A	0.9	45	6.0	*Small* **2023**, 19, 2300683
Meso/Micro-FeNSC	N/A	0.91	62.47	~5.2	*Adv. Mater.* **2023**, 35, 2208942
Fe–1	N/A	0.882	82	6.5	*ACS Nano* **2022**, 16, 15994−16002
P/Fe-N-C	0.99	0.9	N/A	N/A	*J. Am. Chem. Soc.* **2023**, 145, 3647−3655
SA-FeIII/SNPC	0.99	0.91	56	N/A	*Adv. Funct. Mater.* **2023**, 33, 2304277
Fe_{SA}-N-C	0.99	0.9	56	5.75	*Nat. Commun.* **2020**, 11, 2831
Fe-CNG SACs	N/A	0.89	57	~6.0	*Nat. Commun.* **2021**, 12, 5589
Fe_1-$NS_{1.3}C$	0.97	0.86	N/A	N/A	*Angew. Chem., Int. Ed.* **2021**, 60, 25404
FeN_4Cl_1/N	0.96	0.91	35.8	6.0	*Angew. Chem. Int. Ed.* **2021**, 60, 27324−27329

Table S6. Comparison of ZABs performance with other reported transition metal based electrocatalysts ($\triangle E = E_{j=10} - E_{1/2}$).

**Smaple**	**△*E* (V)**	**Open Voltage (V)**		**Peak power density (mW cm⁻²)**		**specific capacity (mAh g⁻¹)**		**Ref**
		**Liquid ZABs**	**Flexible ZABs**	**Liquid ZABs**	**Flexible ZABs**	**Liquid ZABs**	**Flexible ZABs**	
P/Fe-HCBA-2	0.68	1.497	1.46	186	44.5	800.2 @10 mA cm^{-2}	777.1 @1 mA cm^{-2}	this work
FePc||CNTs||NiCo/CP	0.686	1.444	1.37	219.5	85.1	748.2 @10 mA cm^{-2}	N/A	*Adv. Energy Mater.* **2022**, 2202984.
FeP/Fe_2O_3@ NPCA	0.794	N/A	1.428	130.0	40.8	717 @10 mA cm^{-2}	676 @5 mA cm^{-2}	*Adv. Mater.* **2020**, 2002292
$Fe\text{-}P/Cu_3P$-NPC	0.74	1.39	1.41	158.5	70.8	815.3 @10 mA cm^{-2}	N/A	*Small* **2023**, 2301985
ZIF8@FePMPDA920	0.63	1.501	1.4	123.11	N/A	801.2@5 mA cm^{-2}	128.45@1 mA cm^{-2}	*Small* **2023**, 2302464
Fe SA/NCZ	0.68	N/A	1.44	N/A	101	N/A	N/A	*Adv. Funct. Mater.* **2023**, 33, 2213897
FeNi SAs/NC	0.64	N/A	1.45	N/A	42.2	779.4 @5 mA cm^{-2}	N/A	*Adv. Energy Mater.* **2021**, 2101242
Co/N@CNTs/CNMF	0.68	1.52	1.4	133	26.5	777.0 @10 mA cm^{-2}	N/A	*Adv. Funct. Mater.* **2020**, 2003407
Co_2P/Co-NC	0.72	1.45	N/A	187	N/A	837 @10 mA cm^{-2}	N/A	*ACS Appl. Mater. Interfaces* **2023**, 15, 9240–9249
$Co\text{-}CoN_4$@NCNs	0.71	1.468	N/A	118.8	N/A	N/A	N/A	*Adv. Funct. Mater.* **2022**, 32, 2207331
$CoP_3/CeO_2/C$	0.817	1.343	N/A	150	N/A	767.7 @5 mA cm^{-2}	N/A	*Appl. Catal. B: Environ.* **2023,** 321, 122029
$Fe/Ni\text{-}N_x/OC$	0.73	1.525	N/A	148	N/A	712 @5 mA cm^{-2}	N/A	*Adv. Mater.* **2020**, 2004670

3. References

[1] Y. Song, X. Song, X. Wang, J. Bai, F. Cheng, C. Lin, X. Wang, H. Zhang, J. Sun, T. Zhao, H. Nara, Y. Sugahara, X. Li, Y. Yamauchi, *J. Am. Chem. Soc.* **2022**, *144*, 17457–17467.

[2] Y. Chen, S. Ji, Y. Wang, J. Dong, W. Chen, Z. Li, R. Shen, L. Zheng, Z. Zhuang, D. Wang, Y. Li, Angew. Chem. Int. Ed. 2017, 56, 6937–6941.

[3] Y. Chen, C. Wang, Z. Wu, Y. Xiong, Q. Xu, S. Yu, H. Jiang, Adv. Mater. 2015, 27, 5010–5016.

[4] Y. Zhou, R. Lu, X. Tao, Z. Qiu, G. Chen, J. Yang, Y. Zhao, X. Feng, K. Mullen, *J. Am. Chem. Soc.* **2023**, *145*, 3647–3655.

[5] Y. Song, X. Song, X. Wang, J. Bai, F. Cheng, C. Lin, X. Wang, H. Zhang, J. Sun, T. Zhao, H. Nara, Y. Sugahara, X. Li, Y. Yamauchi, *J. Am. Chem. Soc.* **2022**, *144*, 17457–17467.

[6] X. Tang, Y. Wei, W. Zhai, Y. Wu, T. Hu, K. Yuan, Y. Chen, *Adv. Mater.* **2023**, *35*, 2208942.

[7] G. Kresse and J. Hafner, Phys. Rev. B, 1993, 47, 558.

[8] G. Kresse and J. Furthmüller, Comput. Mater. Sci., 1996, 6, 15–50.

[9] P. E. Blöchl, Phys. Rev. B: Condens. Matter Mater. Phys., 1994, 50, 17953.

[10] J. P. Perdew, K. Burke and M. Ernzerhof, Phys. Rev. Lett., 1996, 77, 3865–3868.
